# Scutellarin Alleviates Pulmonary Hypertension by Inhibiting the NF-κB/NLRP3 Signaling Pathway to Suppress Inflammation and Pyroptosis

**DOI:** 10.3390/ph19101595

**Published:** 2026-10-08

**Authors:** Xiaoyu Zhang, Jiahui Xie, Yuan Zhou, Wenting Gui, Jiaxiang Chen, Kai Liu, Runwei Ma

**Affiliations:** 1 Department of Cardiac Surgery, Fuwai Yunnan Hospital, Chinese Academy of Medical Sciences/Affiliated Cardiovascular Hospital of Kunming Medical University, NO. 528 Shahe North Road, Kunming 650000, China; zhangxiaoyu@kmmu.edu.cn (X.Z.); xiejh0509@outlook.com (J.X.); zhouyuan@kmmu.edu.cn (Y.Z.); guiwenting@kmmu.edu.cn (W.G.); gchenjiaxiang@163.com (J.C.); 2 Department of Comprehensive Pediatrics, Kunming Children’s Hospital, NO. 28 Shulin Road, Kunming 650000, China

**Keywords:** pulmonary arterial hypertension, scutellarin, NF-κB signaling pathway, NLRP3 inflammasome, pyroptosis

## Abstract

**Background**: Pulmonary arterial hypertension (PAH) is a progressive fatal cardiovascular disorder characterized by pulmonary vascular remodeling, elevated pulmonary vascular resistance and right heart failure, mainly driven by chronic inflammation and aberrant cell death. Scutellarin (SCU), a natural flavonoid with anti-inflammatory and vasculoprotective properties, exhibits unknown therapeutic effects and mechanisms against PAH. This study aimed to explore the therapeutic potential of SCU for PAH and to clarify its underlying mechanism targeting NF-κB/NLRP3 signaling-mediated inflammation and pyroptosis. **Methods**: Network pharmacology and molecular docking were performed to screen the core therapeutic targets and binding activity of SCU against PAH. A Su5416/hypoxia (SuHx)-induced rat PAH model was established for in vivo verification, and hypoxia-stimulated human pulmonary artery smooth muscle cells (HPASMCs) were used for in vitro functional validation. **Results**: A total of 100 overlapping targets of SCU and PAH were identified, with core hub targets enriched in inflammation and pyroptosis pathways, and stable binding between SCU and core targets was verified. SCU significantly improved right ventricular function and pulmonary hemodynamics, alleviating pulmonary vascular remodeling and perivascular fibrosis in PAH rats. Mechanically, SCU blocked NF-κB/NLRP3 signaling, inhibited cell pyroptosis and inflammatory cytokine release, and downregulated vascular remodeling-related protein expression. Additionally, SCU dose-dependently suppressed the hypoxia-induced proliferation and pyroptosis of HPASMCs. **Conclusions**: SCU ameliorates PAH-induced pulmonary vascular remodeling and right ventricular dysfunction via inhibiting the NF-κB/NLRP3 inflammatory and pyroptosis signaling cascade, which supports its potential as a promising natural candidate agent for PAH treatment.

## 1. Introduction

PAH is a devastating cardiovascular disorder pathologically characterized by progressive pulmonary vascular remodeling, elevated pulmonary vascular resistance and subsequent right heart failure [1]. The pathological mechanisms underlying PAH involve the excessive proliferation and apoptosis resistance of pulmonary artery smooth muscle cells, sustained inflammatory activation, aberrant cell death and excessive extracellular matrix deposition [2,3]. Although substantial advances have been achieved in clinical therapeutic strategies, the long-term prognosis of PAH patients remains unsatisfactory owing to its intricate and multifactorial pathogenesis [4,5]. Cumulative studies have demonstrated that vascular inflammation serves as a pivotal driver during PAH initiation and progression [6,7], yet the precise molecular mechanisms remain incompletely defined. Accordingly, developing novel safe and effective therapeutic agents targeting inflammatory and cell death pathways carries profound clinical significance.

The NF-κB signaling cascade acts as a master transcriptional regulator of inflammatory genes. Upon activation, NF-κB translocates into the nucleus to upregulate the expression of NLRP3, pro-IL-1β and pro-IL-18. The assembled NLRP3 inflammasome further facilitates caspase-1 cleavage and triggers gasdermin D (GSDMD)-dependent pyroptosis, which ultimately triggers robust secretion of pro-inflammatory cytokines and exacerbates vascular inflammatory injury [8,9]. Mounting evidence has validated that blockade of the NF-κB/NLRP3-pyroptosis axis markedly alleviates pulmonary vascular remodeling and ameliorates PAH pathological phenotypes [10].

SCU, a natural flavonoid extracted from Erigeron breviscapus (Vant.) Hand.-Mazz., has been widely applied for the management of cardiovascular and cerebrovascular disorders, exhibiting prominent anti-inflammatory, anti-fibrotic and vasculoprotective bioactivities [11,12,13]. Accumulating preclinical research has revealed that SCU suppresses NF-κB activation and NLRP3 inflammasome-triggered pyroptosis across multiple disease models [14,15]. Nevertheless, whether SCU exerts protective effects against PAH via modulating the NF-κB/NLRP3 signaling axis remains to be fully elucidated.

In the present work, we integrated network pharmacology, molecular docking, in vivo SuHx-induced PAH rat models and in vitro cellular experiments to systematically characterize the therapeutic efficacy and underlying molecular mechanisms of SCU against PAH. We hypothesize that SCU restrains inflammatory response and pyroptosis by inhibiting the NF-κB/NLRP3 signaling cascade, thereby mitigating the PAH lesions and right ventricular dysfunction resulting from severe pulmonary vascular remodeling.

Despite accumulating evidence supporting the anti-inflammatory and vasculoprotective activities of SCU in multiple cardiovascular diseases, its protective function and concrete molecular mechanism in regards to PAH have not been fully elucidated. The present study fills this knowledge gap by combining computational prediction and multi-level experimental verification. The novelty of this work lies in the revelation that SCU ameliorates PAH-associated pulmonary vascular remodeling and right-ventricular dysfunction through suppressing NF-κB/NLRP3-driven inflammation and pyroptosis. These findings offer new mechanistic insights for understanding natural-compound-mediated PAH intervention and provide a pre-clinical experimental basis for the translational development of SCU as a potential therapeutic candidate for PAH.

## 2. Results

### 2.1. SCU Targeted Gene Networks and Pathways in PAH

**Figure 1:**
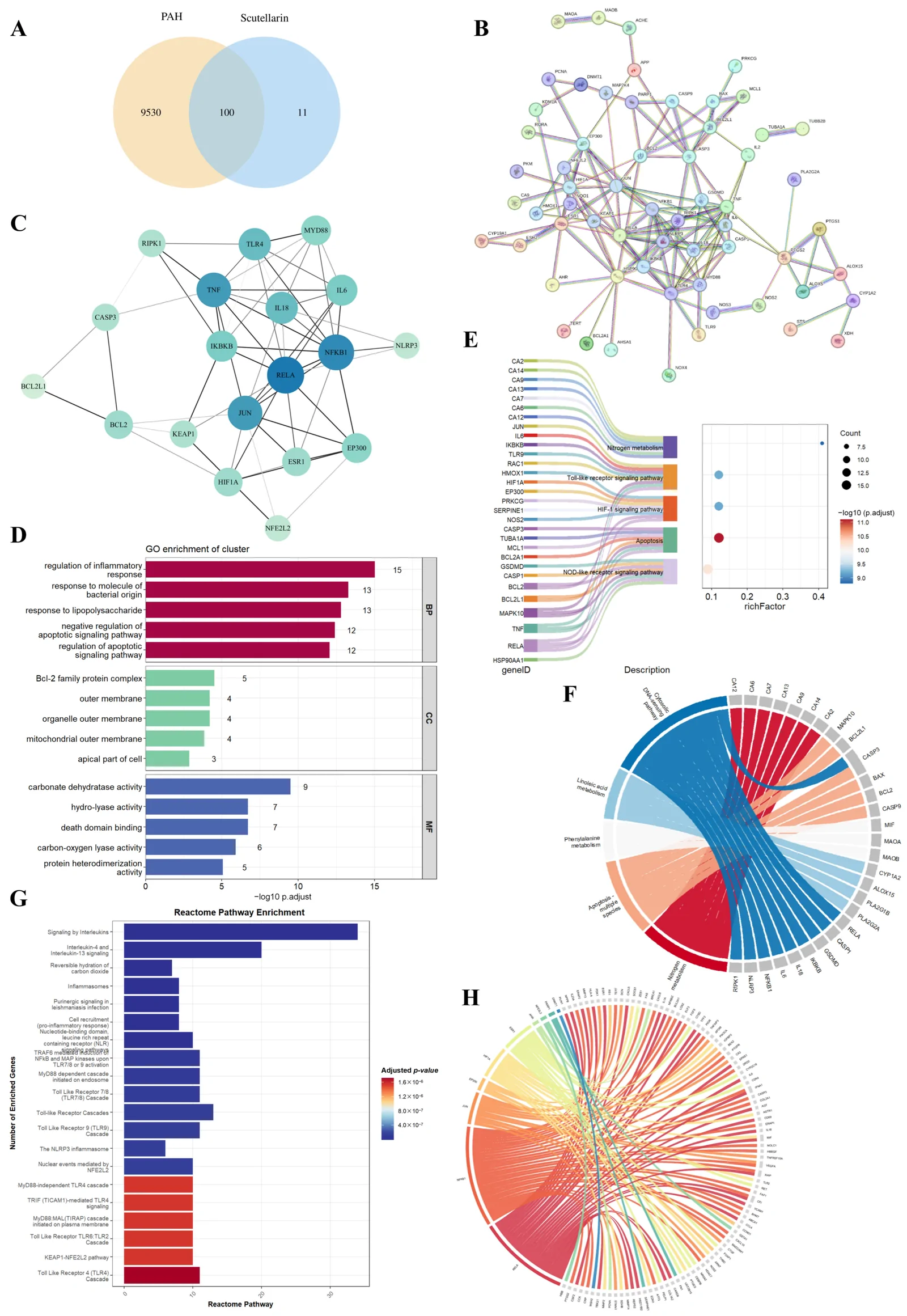
Identification, functional enrichment analysis, and PPI network construction of overlapping targets between SCU and PAH. (**A**) Venn diagram showing the overlap between SCU-related targets and PAH-associated genes. (**B**) PPI network of the overlapping targets. Nodes represent proteins, and edges indicate interactions between them, illustrating molecular relationships and key signaling connections. (**C**) Core module analysis of the PPI network. Hub genes were identified based on topological parameters, including degree and betweenness centrality. Dark blue nodes represent the core hub proteins in the network. (**D**) GO enrichment analysis. The length of the bars represents the enrichment significance (−log10 adjusted *p*-value), and the numbers indicate the count of enriched genes. (**E**) KEGG pathway enrichment analysis. The left panel is a Sankey diagram showing the relationships between genes and their corresponding pathways; the right panel is a bubble plot, where bubble size corresponds to the number of enriched genes, and color intensity represents the adjusted *p*-value. (**F**) Circular plot of KEGG disease pathway enrichment. The outer bars represent enriched genes, and the inner lines connect each gene to its corresponding disease pathway. (**G**) Bar plot of Reactome pathway enrichment. The color gradient from blue to red indicates increasing adjusted *p*-values. (**H**) Circular plot of TF enrichment analysis. The outer bars represent TFs, and the inner lines denote the regulatory relationships between TFs and their target genes.

To elucidate the molecular mechanism by which SCU intervenes in PAH, a total of 9530 PAH-related targets (yellow region) and 111 SCU associated targets (blue region) were retrieved from public databases. Venn diagram analysis identified 100 overlapping core genes (Figure 1A).

We note that identification of overlapping genes via Venn-diagram analysis alone cannot establish direct functional causality. This intersection analysis was applied primarily to filter candidate downstream effectors modulated by SCU that may counteract PAH-related differential gene alterations, providing directional computational hints for subsequent enrichment, molecular docking, and experimental validation rather than serving as conclusive mechanistic proof.

A PPI network was subsequently constructed based on these intersecting genes, and topological analysis was performed to screen hub targets. Multiple pivotal hub genes, including RELA, JUN, NLRP3, TNF, and IL18, were ultimately recognized (Figure 1B). A core subnetwork was further established (Figure 1C), and the results demonstrated that RELA, JUN, NLRP3, TNF, and IL18 were centrally located, with intensive interactive correlations. In particular, transcription factors (TFs) represented by RELA and JUN, as well as inflammation- and pyroptosis-related molecules such as NLRP3 and IL18, served as critical regulatory hubs within the network. These findings suggested that SCU may exert its therapeutic effects predominantly by modulating inflammation and pyroptosis-related signaling cascades.

GO enrichment analysis indicated that the overlapping targets were mainly involved in the regulation of inflammatory responses, apoptotic signaling pathways, and NLRP3 inflammasome-related biological processes (Figure 1D). Pathway enrichment analysis further confirmed that the targets of SCU were significantly enriched in the NOD-like receptor signaling pathway and the NLRP3 inflammasome pathway (Figure 1E,G), along with the apoptotic signaling pathway. Key pyroptosis-execution molecules, including GSDMD and CASP1, were also integrated into the core regulatory network (Figure 1F,H).

Taken together, the above results indicate that SCU alleviates PAH-associated pathological events (inflammation and pyroptosis) via synergistic regulation of the pivotal transcription factors TFs NF-κB, JUN and HIF1A.

### 2.2. Physicochemical, ADME and Molecular Docking Analysis of the Effects of SCU Against Core PAH Targets

We first characterized the physicochemical properties, drug-likeness and ADME profiles of SCU using the SwissADME platform. Drug-likeness prediction showed that SCU had a drug-likeness score of 1.0. Its molecular formula was C_21_H_18_O_12_, with a molecular weight of 462.36 g/mol and a topological polar surface area (TPSA) of 207.35 Å. Structural parameter analysis identified four rotatable bonds, twelve hydrogen bond acceptors and seven hydrogen bond donors within the SCU skeleton. Pharmacokinetic assessment indicated that SCU displays weak gastrointestinal absorption and lacks blood-brain barrier permeability. Moreover, SCU serves as a substrate of P-glycoprotein (P-gp) but shows no inhibitory activity against cytochrome P450 (CYP) isoforms. Detailed information on the interacting residues/atoms, hydrogen-bond numbers, and hydrogen-bond lengths is summarized in Table 1.

**Figure 2:**
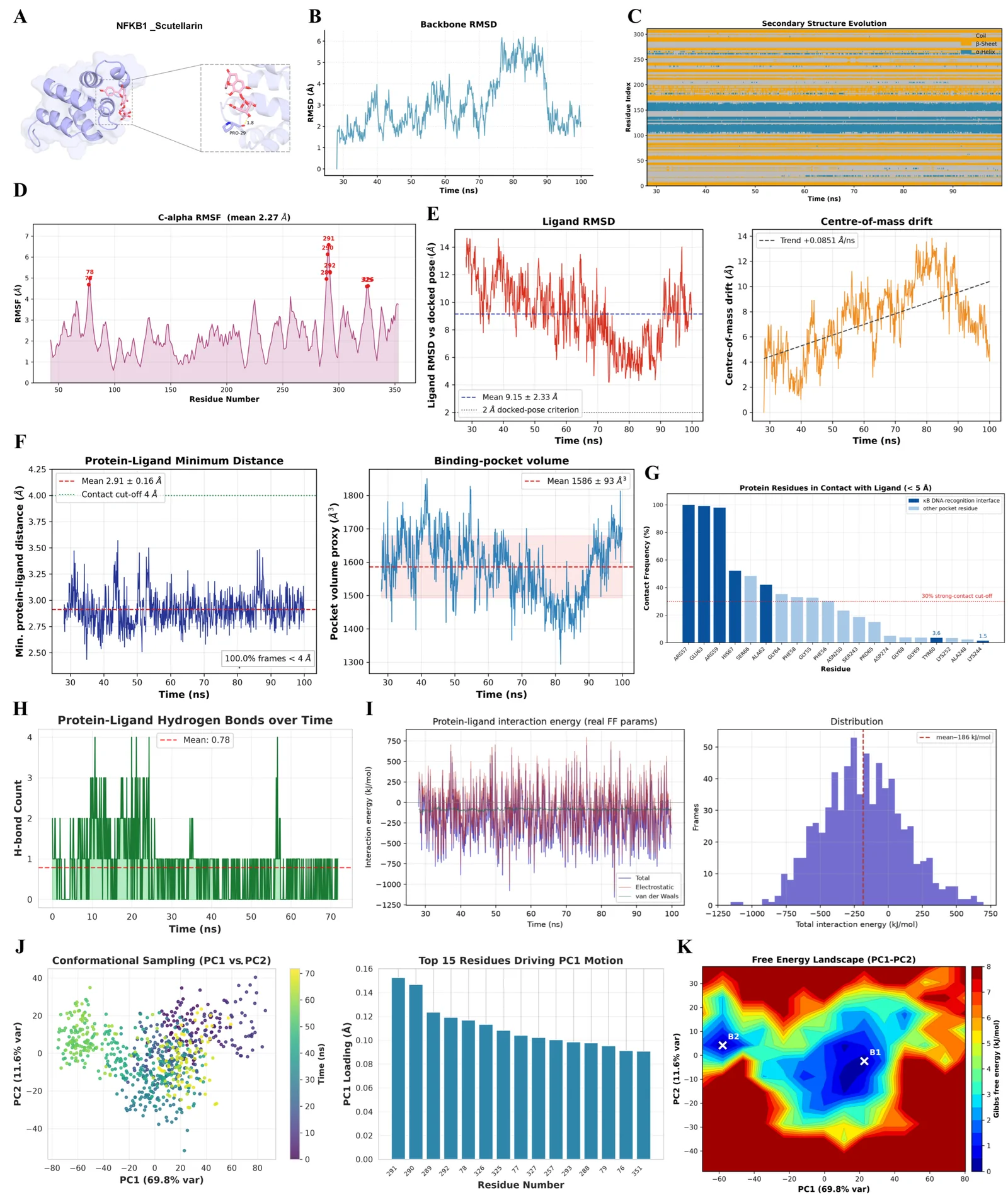
Molecular docking analysis and molecular dynamics simulation analysis of SCU-scutellarein NF-κB p50 complex. (**A**) Molecular docking simulations illustrating the binding interactions between SCU (red stick representation) and the target proteins NFKB1. (**B**) Time evolution of backbone RMSD of NF κB p50 protein during 100 ns MD simulation. (**C**) Residue-wise DSSP secondary structure time series. (**D**) Cα RMSF profile of the protein, with the major fluctuation peaks labeled (residues 78, 77, 289, 290, 291, 292, 325, and 326). (**E**) Time-dependent changes in ligand heavy-atom RMSD and center-of-mass displacement relative to the initial docking conformation. (**F**) Time-dependent changes in the minimum distance between the ligand and binding pocket (left) and pocket volume (right). (**G**) Protein–ligand contact residue profile (5 Å cutoff; top 20 residues ranked by occupancy; red dashed line indicates the 30% threshold for strong contacts, and dark blue bars denote residues within the κB DNA recognition interface). (**H**) Time-dependent changes in the number of protein–ligand hydrogen bonds. (**I**) Direct protein–ligand interaction energies (left: time-dependent profiles of electrostatic, van der Waals, and total energies; right: histogram of the total energy distribution). (**J**) Principal component analysis (PCA) (left: PC1–PC2 projection scatter plot colored according to simulation time; right: the top 15 residue loadings contributing to PC1). (**K**) PC1–PC2 free energy landscape (FEL), showing two major identified basins (B1 and B2).

Subsequently, molecular docking was implemented to quantify the binding affinity of SCU to five core hub PAH targets (NFKB1, RELA, JUN, TNF and IL18), with all binding free energies (ΔG) listed in Table 1. SCU displayed the tightest predicted binding interaction for the JUN-DNA complex (PDB ID: 1JNM), with a ΔG value of −9.4 kcal/mol. Notably, the top-ranked docking pose revealed that SCU forms hydrogen bonds mainly with deoxyribonucleotide residues of the double-stranded DNA within this JUN-DNA crystal rather than with amino-acid residues of the JUN protein. This in silico prediction raises the hypothetical possibility that SCU may interfere with JUN-dependent transcription by interacting with AP-1-recognition DNA; nevertheless, this observation requires further experimental verification. Following JUN, NFKB1 and IL18 presented equal ΔG levels of −7.1 kcal/mol. PyMOL software (version 3.0.3) was utilized to visualize the optimal binding poses of SCU with each target macromolecule (Figure 2A and Figure S1). Further structural characterization verified that SCU formed extensive hydrogen bonds with all target macromolecules across all docking conformations, which markedly stabilized the ligand-receptor complexes.

### 2.3. Molecular Dynamics Analysis of the SCU-NF-κB Complex

To validate the dynamic stability of the SCU-NF-κB p50 complex predicted by molecular docking, a 100 ns all-atom molecular dynamics (MD) simulation was performed. The protein backbone reached conformational equilibrium at approximately 28 ns, with RMSD fluctuations stably maintained at 2–6 Å in subsequent trajectories (Figure 2B). The time-averaged C-α RMSD and radius of gyration (Rg) were 3.02 ± 1.26 Å (0.00–6.19 Å) and 25.70 ± 0.29 Å (24.91–26.57 Å), respectively (Figure S2). The protein exhibited a stable time-averaged solvent-accessible surface area (SASA) of 17 090 ± 212 Å^2^ throughout the simulation (Figure S3). Residue-wise DSSP analysis confirmed stable β-sheets and α-helices in the DNA-binding, dimerization, and insertion domains, with no local unfolding events, consistent with RMSD and Rg stability results (Figure 2C).

To characterize the flexible regions within the complex and evaluate the structural rigidity of the docking site, Cα RMSF analysis was performed. The average Cα RMSF over the entire trajectory was 2.27 Å (range: 0.59–6.59 Å). The most prominent fluctuations were mainly localized to two loop regions within the DNA-binding domain: the Arg289–Gly292 loop (maximum RMSF: 6.6 Å) and the Gly77–Gly78 loop (5.0 Å). The dimerization domain (residues 255–353) exhibited relatively higher overall mobility, with residues 325–326 showing an RMSF value of 4.6 Å. Notably, key DNA interface residues directly involved in hydrogen bonding with the ligand, including Arg57, Arg59, Phe56, Phe58, and Tyr60, maintained low RMSF values (<2.6 Å), indicating that the ligand-binding interface remained highly rigid and structurally stable throughout the simulation (Figure 2D).

Using the initial docking pose as a reference, ligand heavy-atom RMSD and center-of-mass displacement were analyzed during the 100 ns simulation (Figure 2E). The ligand RMSD remained high (9.15 ± 2.33 Å; range: 4.17–14.66 Å), with a center-of-mass displacement of 7.42 ± 2.54 Å. No frames showed RMSD values below 2 or 3 Å, indicating that the initial docking pose was not maintained and the ligand underwent repositioning toward a stable secondary site. Clustering analysis using a 3 Å RMSD cutoff identified 36 binding modes, with the five most populated clusters accounting for 19.9%, 12.9%, 8.8%, 7.6%, and 5.7% of the trajectory, respectively. Despite continuous ligand reorientation, stable pocket engagement was maintained, as indicated by a minimum ligand–pocket distance of 2.91 ± 0.16 Å and 100% occupancy of contacts within 4 Å. These results suggest dynamic exchange among multiple energetically similar microstates rather than ligand dissociation.

The minimum ligand-pocket distance and pocket volume were monitored throughout the simulation (Figure 2F). The ligand maintained persistent pocket engagement, with a minimum distance of 2.91 ± 0.16 Å (2.43–3.57 Å) and 100% occupancy of contacts within 4 Å. The pocket volume remained highly stable (1,586 ± 93 Å^3^; range: 1,294–1,851 Å^3^), with only ~6% fluctuation, indicating that ligand repositioning occurred within a structurally conserved pocket without significant expansion or collapse of the binding interface.

Residue–ligand contact frequencies during the production phase were analyzed using a 5 Å cutoff (Figure 2G). Ten residues showed strong contacts with occupancy ≥30%, among which ARG57 (100.0%), GLU63 (99.3%), and ARG59 (98.1%) maintained persistent interactions, forming an “anchor triangle” at the binding interface. Additional key contacts included HIS67, SER66, ALA62, GLY64, PHE58, GLY55, and PHE56. Notably, seven residues within the κB DNA recognition interface contacted the ligand, with ARG57, GLU63, ARG59, HIS67, and ALA62 meeting the strong-contact criterion, whereas TYR60 and LYS244 exhibited only transient interactions. These findings indicate substantial overlap between the ligand-binding site and the κB DNA recognition interface, suggesting that scutellarein may inhibit NF-κB activity through competitive occupation of the DNA-binding surface.

Protein–ligand hydrogen bonds were analyzed using D–A distance <3.5 Å and D–H···A angle >150° criteria (Figure 2H; Table S4). The average number of hydrogen bonds per frame was 0.78 ± 0.83 (range: 0–4), with 85.3% of frames containing 0–1 hydrogen bonds, indicating a sparse but persistent hydrogen-bond network. A total of 31 hydrogen-bond patterns were identified, with ARG57/NH1···Lig/O8x (31.3%), ARG57/N···Lig/O8x (17.2%), Lig/O10x···GLY55/O (6.5%), and ARG59/NH1···Lig/O8x (6.0%) as the dominant interactions. ARG57 contributed ~48.5% of total hydrogen-bond occupancy through two interactions, serving as a key hydrogen-bond anchor. The ligand O8x atom (4′-OH/5-OH region) acted as the primary acceptor for ARG-mediated electrostatic hydrogen bonding. Although HIS67 showed high contact occupancy (52.3%), its corresponding hydrogen bond occupancy was only 0.3%, suggesting a predominant role through π-stacking and hydrophobic interactions rather than hydrogen bonding. Using the actual AMBER14SB/GAFF2-AM1BCC force-field parameters implemented in work/system.xml and a 1.2 nm cutoff, we further evaluated the direct protein-ligand interaction energies (Figure 2I). The χ1 dihedral angle flipping profile of pocket residues revealed that core binding residues (ARG57, ARG59, PHE56, PHE58, and TYR60) maintained stable side-chain orientations with no χ1 flips during the 100 ns simulation. In contrast, peripheral residues, including LYS80, LEU70, ASN139, and ASN250, showed substantially higher side-chain flexibility (50, 26, 22, and 19 flips, respectively). Together with the contact analysis, these results indicate a rigid core binding interface surrounded by dynamically adaptable peripheral residues (Figure S5).

After mean-centering the protein Cα coordinates, principal component analysis (PCA) was performed, and the free energy landscape (FEL) was constructed by Boltzmann inversion of the PC1–PC2 projection (14 × 14 bins) (Figure 2J). PC1 and PC2 accounted for 69.8% and 11.6% of the variance, respectively, capturing 81.4% of the overall conformational fluctuations. Two major basins were identified: B1 (PC1 ≈ +23 Å, PC2 ≈ −2 Å), representing the dominant state sampled throughout 28.1–99.0 ns (229 frames, 32%), and B2 (PC1 ≈ −58 Å, PC2 ≈ +4 Å), a transient state accessed only during 75.9–89.0 ns and corresponding to the RMSD elevation period. The small energy difference between B1 and B2 (0.11 kJ/mol, below RT ≈ 2.49 kJ/mol) does not support thermodynamic ranking of the states but indicates the presence of multiple accessible conformational states. The maximum FEL value (7.93 kJ/mol) represents unsampled regions rather than a true energy barrier (Figure 2K). Dynamic cross-correlation matrix (DCCM) analysis is presented in Figure S6 and Table S5.

### 2.4. SCU Ameliorates Right Ventricular Function and Attenuates Pulmonary Vascular Fibrosis in PAH Rats

**Figure 3:**
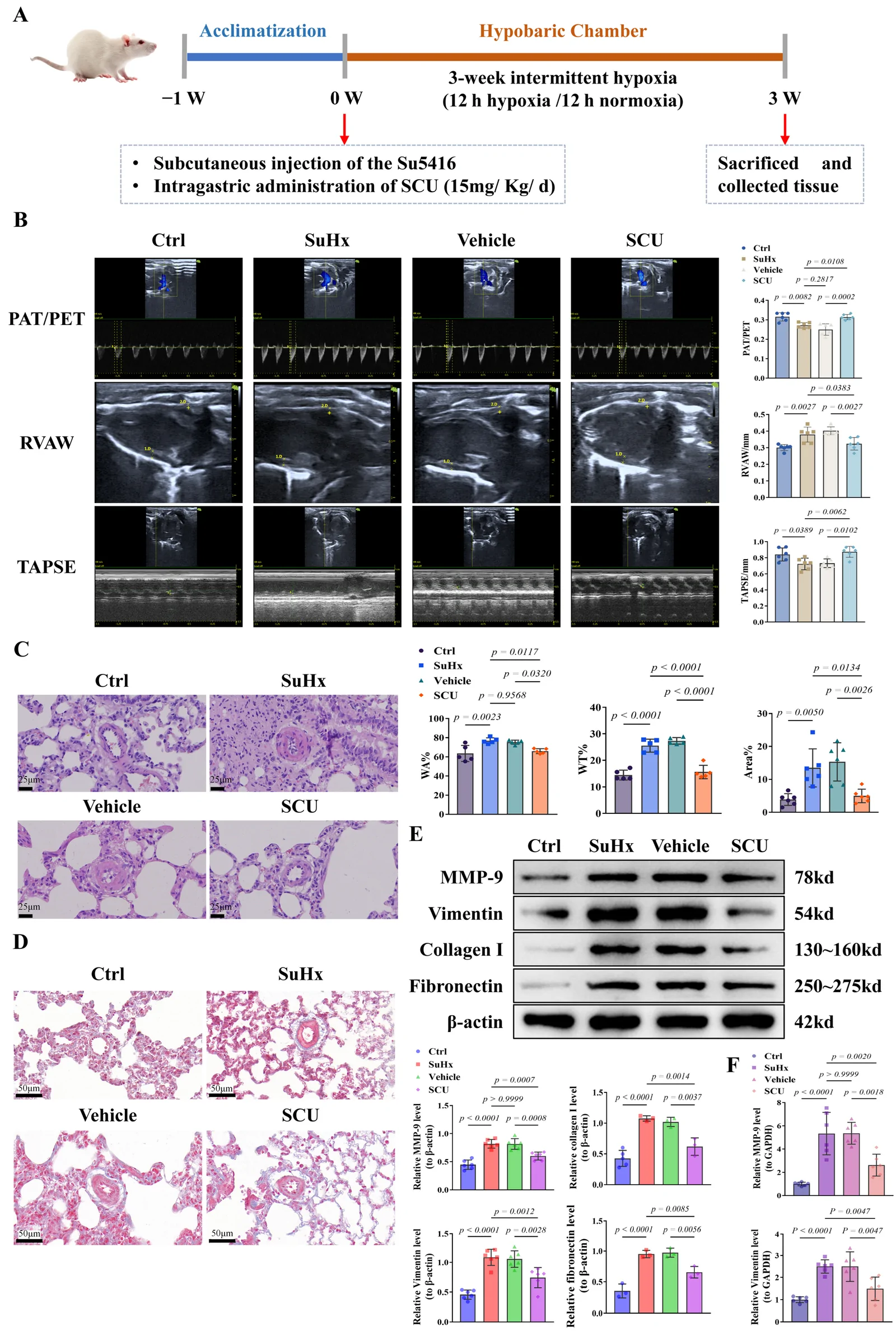
Therapeutic effects of SCU on cardiac function, pulmonary vascular remodeling, and fibrosis in SuHx-induced PAH rats. (**A**) Experimental design of the animal model; created in BioRender, Xiaoyu Zhang, (2026); httpss://BioRender.com/er4zeye. (**B**) Representative echocardiographic images and quantitative analyses of cardiac function and right ventricular (RV) remodeling in each group (Ctrl, SuHx, Vehicle, and SCU). Parameters include PAT/PET, RVAW, and TAPSE, indicating pulmonary hemodynamics and RV systolic function. (**C**) Representative HE staining of lung tissues showing morphological changes in pulmonary arterioles. Quantitative analysis of vascular remodeling, including WT% and WA%, is presented. Scale bar = 25 μm. (**D**) Representative Masson’s trichrome staining of lung tissues showing perivascular collagen deposition and fibrosis. Quantitative analysis of fibrotic remodeling is shown as collagen area percentage. Scale bar = 50 μm. (**E**) Representative Western blot images and quantitative analysis of MMP-9, vimentin, collagen I and fibronection protein expression in lung tissues. β-actin was used as the loading control. (**F**) Relative mRNA expression levels of MMP-9 and vimentin determined by qRT-PCR. GAPDH served as the internal reference gene. Data are presented as mean ± SD. *p*-values represent intergroup statistical differences. SCU: scutellarin; SuHx: SU5416 combined with hypoxia; Ctrl: control; PAT: pulmonary artery acceleration time; PET: pulmonary artery ejection time; TAPSE: tricuspid annular plane systolic excursion; RVAW: right ventricular anterior wall thickness.

To investigate the therapeutic efficacy of SCU against PAH, a rat PAH model was established via combined SU5416 injection and chronic hypoxia exposure (Figure 3A). Echocardiography was performed to assess cardiac function and hemodynamic parameters across all experimental groups (Figure 3B). Compared with the control (Ctrl) group, the SuHx model group exhibited a markedly decreased PAT/PET, whereas SCU treatment significantly restored this ratio, demonstrating its capacity to ameliorate PAH-related hemodynamic disturbances. Further right ventricular functional examinations revealed increased RVAW and reduced TAPSE in SuHx rats, indicative of right ventricular hypertrophy and impaired systolic performance. SCU administration robustly reversed these pathological abnormalities and rescued right ventricular systolic function. No statistically significant differences in phenotypic indices were detected between the vehicle group and the control group (*p* > 0.05), which validated the prominent cardioprotective potency of SCU.

Hematoxylin and eosin (HE) staining of lung tissue sections (Figure 3C) revealed pronounced structural disruption of pulmonary arterioles in SuHx rats, characterized by marked vascular wall thickening and luminal narrowing, which are hallmark pathological features of pulmonary vascular remodeling. Quantitative morphometric analysis demonstrated that the percentage of vascular wall thickness (WT%) and vascular wall area (WA%) were drastically elevated in model rats; SCU treatment remarkably suppressed aberrant pulmonary vascular wall proliferation and alleviated pathological vascular remodeling.

Masson’s trichrome staining (Figure 3D) demonstrated massive collagen deposition and severe perivascular fibrosis within the pulmonary tissue of SuHx rats. Quantitative statistical analysis further verified that SCU substantially attenuated collagen accumulation and shrank fibrotic lesions, thereby effectively mitigating pulmonary vascular fibrotic injury.

To further explore the effects of SCU on pulmonary vascular remodeling, we examined the expression of extracellular matrix degradation and mesenchymal transition-related markers in SuHx-induced PAH lung tissues.

Western blot analysis demonstrated that, compared with the Ctrl group, the SuHx and Vehicle groups exhibited significantly increased protein expression of MMP-9, vimentin, collagen I and fibronectin. In contrast, SCU treatment markedly reduced the expression of MMP-9, vimentin, collagen I and fibronectin, indicating attenuation of matrix remodeling and mesenchymal activation. Quantitative analysis further confirmed that SCU significantly reversed SuHx-induced upregulation of MMP-9 and vimentin (Figure 3E).

Consistent with the protein data, qRT-PCR results (Figure 3F) showed that mRNA levels of MMP-9 and vimentin were significantly elevated in the SuHx and Vehicle groups compared with the results for the Ctrl group. SCU administration significantly suppressed these increases, suggesting transcriptional regulation of remodeling-associated genes.

### 2.5. Regulatory Effects of SCU on the NF-κB/NLRP3 Signaling Pathway in a Rat Model of PAH

**Figure 4:**
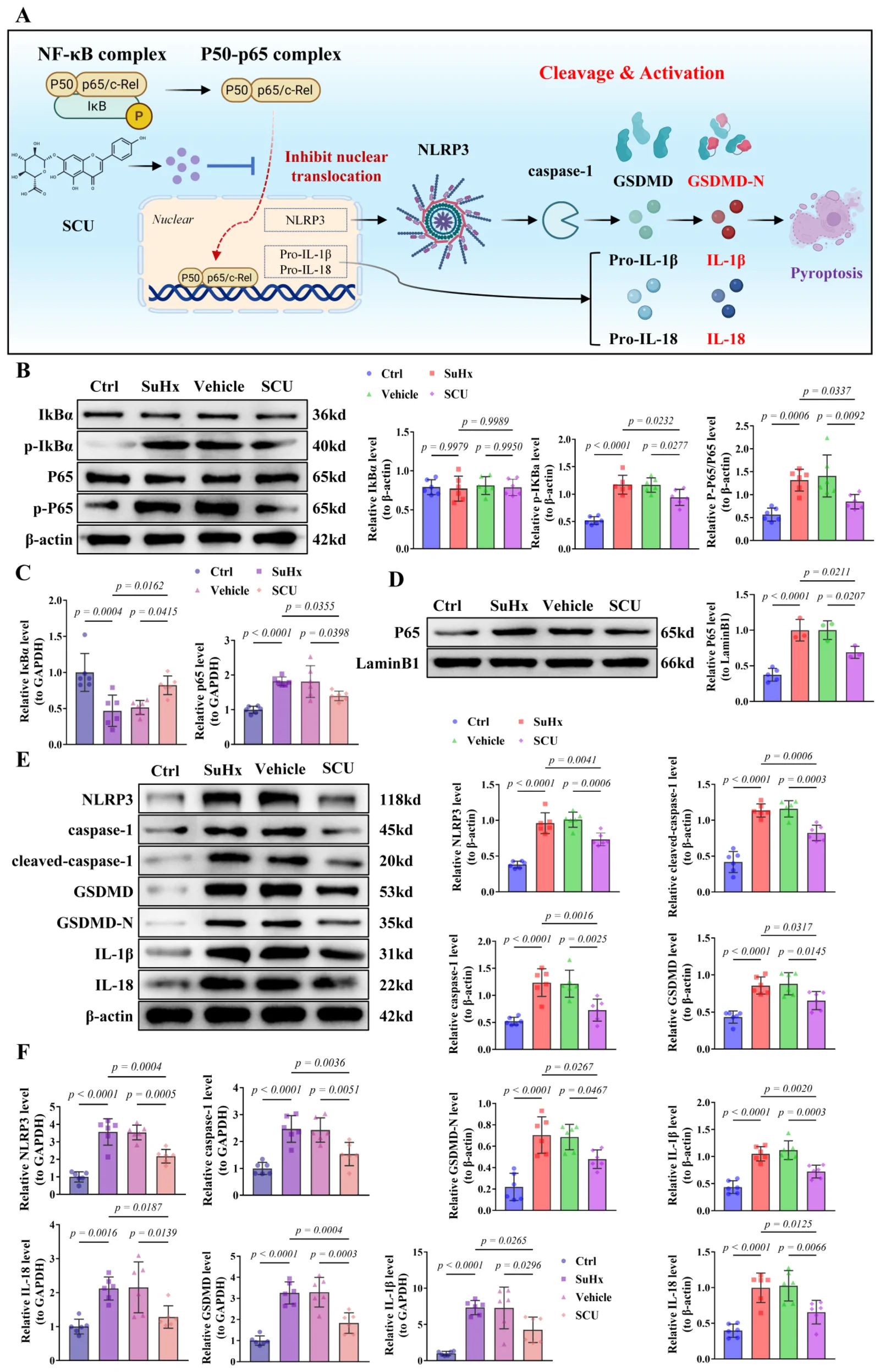
SCU represses the NF-κB signaling pathway and NLRP3-dependent pyroptosis in the SuHx-rat pulmonary hypertension model. (**A**) Working model depicting how SCU blocks NF-κB p65 nuclear translocation, restrains NLRP3 inflammasome assembly, and thereby alleviates pyroptotic cell death. Created in BioRender, Xiaoyu Zhang, (2026); https://BioRender.com/7vykjaw. (**B**,**C**) Immunoblotting and densitometric quantification of IκBα, phosphorylated-IκBα, P65 and phosphorylated-P65 in whole-tissue lysates. (**D**) Nuclear-protein immunoblot showing nuclear P65 abundance, with LaminB1 as the nuclear internal reference. (**E**,**F**) Immunoblot analysis and quantitative measurement of NLRP3 inflammasome-pyroptosis-related proteins, including NLRP3, caspase-1, cleaved-caspase-1, GSDMD, GSDMD-N, IL-1β and IL-18. Data are presented as mean ± SD. *p*-values represent intergroup statistical differences. SCU: scutellarin; SuHx: SU5416 combined with hypoxia; Ctrl: control.

To determine whether SCU participates in the regulation of the NF-κB/NLRP3 signaling cascade during PAH progression (Figure 4A), we examined the protein and mRNA expression levels of key NF-κB-related molecules, including IκBα and p65. In addition, the nuclear localization of p65 was assessed.

Western blot analysis was performed to evaluate the protein expression levels of total IκBα, phosphorylated IκBα (p-IκBα), total p65, and phosphorylated p65 (p-p65) (Figure 4B). The results showed that no significant differences were observed in the relative expression levels of total IκBα among the experimental groups (all *p* > 0.05). Compared with the Ctrl group, the SuHx group exhibited a significant increase in the p-p65/p65 ratio (*p* = 0.0006). Following SCU treatment, the elevated p-p65/p65 ratio was markedly reduced (SuHx vs. SCU, *p* = 0.0092), indicating that SCU effectively suppresses p65 phosphorylation and inhibits NF-κB pathway activation.

Consistent with the protein-level findings, qPCR analysis further revealed transcriptional changes in IκBα and p65 expression (Figure 4C). Compared with the Ctrl group, IκBα mRNA expression was significantly decreased in SuHx rats (*p* = 0.0004), while no significant difference was observed between the Vehicle and SuHx groups (*p* > 0.9999). SCU administration significantly restored IκBα transcriptional levels (SuHx vs. SCU, *p* = 0.0415). Regarding p65 mRNA expression, the SuHx group showed a marked upregulation compared with the results for the Ctrl group (*p* < 0.0001), whereas SCU treatment significantly reversed this increase (SuHx vs. SCU, *p* = 0.0398).

Furthermore, nuclear p65 expression was examined to evaluate NF-κB nuclear translocation. The results demonstrated that SCU treatment significantly decreased the accumulation of p65 in the nucleus, suggesting that SCU suppresses NF-κB activation by inhibiting p65 nuclear translocation (Figure 4D).

To investigate whether SCU regulates NLRP3 inflammasome-mediated pyroptosis, the downstream event of NF-κB signaling in SuHx-induced pulmonary hypertension, we systematically analyzed key molecules of this pathway. As illustrated in the schematic diagram in Figure 4A, activation of the NLRP3 inflammasome promotes the cleavage-dependent activation of caspase-1 and the proteolytic processing of GSDMD, driving the maturation and release of IL-1β and IL-18 and ultimately triggering pyroptosis.

Western blot analysis (Figure 4E) revealed that, compared with the Ctrl group, the SuHx and Vehicle groups exhibited significantly increased protein expression levels of NLRP3, cleaved caspase-1, GSDMD, GSDMD-N, IL-1β, and IL-18. However, SCU intervention markedly attenuated these elevated protein levels, indicating that SCU inhibits inflammasome activation and downstream pyroptotic signaling.

Consistently, qRT-PCR analysis (Figure 4F) demonstrated that SCU treatment significantly reduced the SuHx-induced transcriptional upregulation of NLRP3, caspase-1, GSDMD, IL-1β, and IL-18.

Collectively, these findings indicate that SCU suppresses NF-κB/NLRP3 signaling activation, inhibits NLRP3 inflammasome-mediated caspase-1-dependent pyroptosis, and thereby reduces inflammatory cytokine production in SuHx-induced PAH.

### 2.6. Dose-Dependent Inhibitory Effects of SCU on Hypoxia-Induced Proliferation and Inflammatory Activation in HPASMCs

To validate the protective effect of SCU on pulmonary artery smooth muscle cells under a hypoxic microenvironment, we constructed an in vitro hypoxia (HYP) cell model and treated cells with gradient concentrations of SCU (5, 10, 20, 50 μM).

**Figure 5:**
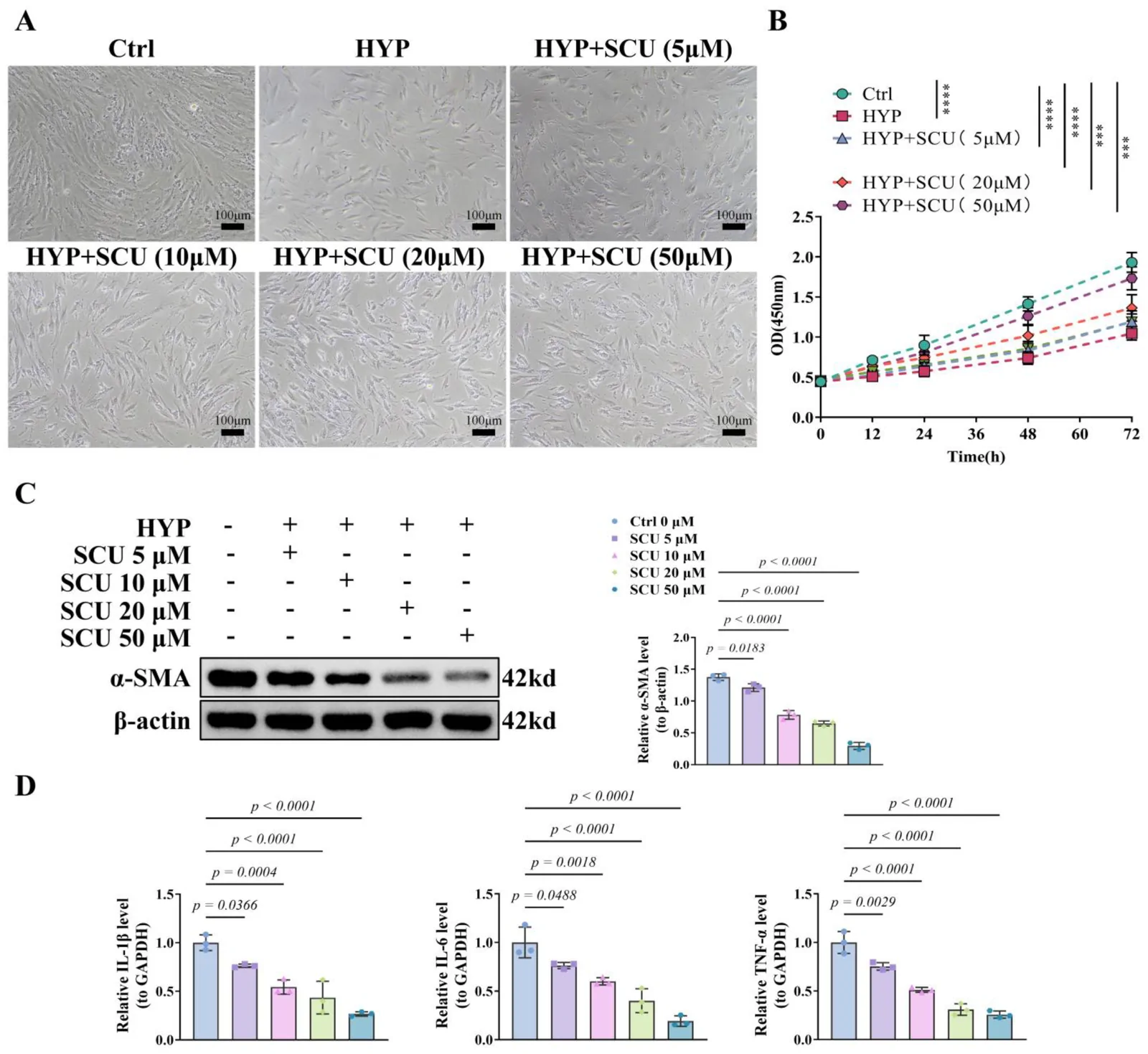
Dose-dependent effects of SCU on hypoxia-induced proliferation dysregulation and inflammatory activation in HPASMCs. (**A**) Representative bright-field images of cells in each group; scale bar = 100 μm. (**B**) CCK-8 assay showing cell proliferation viability curves (OD450 nm) over 0–72 h. (**C**) Western blot analysis of the smooth muscle contractile marker α-SMA, including representative bands and quantitative results; β-actin was used as the loading control. (**D**) qRT-PCR analysis of mRNA expression levels of the pro-inflammatory cytokines IL-1β, IL-6, and TNF-α; GAPDH was used as the internal reference gene. Data are presented as mean ± SD. *p*-values represent intergroup statistical differences. HYP: hypoxia treatment; SCU: scutellarin; Ctrl: normoxic control group; OD: optical density. *** *p* < 0.001, **** *p* < 0.0001.

Cell morphological observation under an inverted microscope (Figure 5A) revealed that HPASMCs in the Ctrl group presented a regular spindle morphology. After hypoxic stimulation, cells exhibited abnormal hypertrophic morphology and disordered arrangement. Intervention with gradient concentrations of SCU dose-dependently restored the normal spindle shape of hypoxic HPASMCs.

A CCK-8 proliferation assay was performed to monitor cell viability at 0, 12, 24, 36, 48, 60 and 72 h (Figure 5B). Hypoxia significantly accelerated the proliferation of HPASMCs compared with the results for the Ctrl group at all time points (*p* < 0.001). Co-treatment with SCU remarkably inhibited hypoxia-triggered cell overproliferation in a concentration-dependent manner; the inhibitory effect was gradually enhanced with the increase in SCU concentration from 5 μM to 50 μM.

α-SMA is a canonical marker of the contractile phenotype of pulmonary artery smooth muscle cells, and its downregulation indicates pathological phenotypic switching to the synthetic phenotype. Western blot results (Figure 5C) demonstrated that hypoxia markedly reduced the relative expression of α-SMA (*p* < 0.0001). SCU administration dose-dependently reversed the hypoxia-induced decline in α-SMA protein, with significant differences observed in all SCU concentration groups vs. the HYP (all *p* < 0.0001).

qPCR was further applied to detect the transcription levels of the pro-inflammatory cytokines IL-1β, IL-6 and TNF-α (Figure 5D). Compared with the resutls for the Ctrl group, hypoxia drastically upregulated the mRNA expression of IL-1β, IL-6 and TNF-α (all *p* < 0.0001). SCU treatment significantly suppressed the transcription of these three inflammatory factors in a concentration-dependent manner. Statistical differences between the HYP group and each SCU intervention group were as follows: IL-1β (5 μM: *p* = 0.0366, 10/20/50 μM: *p* < 0.0001), IL-6 (5 μM: *p* = 0.0488, 10/20/50 μM: *p* < 0.0001), and TNF-α (5 μM: *p* = 0.0029, 10/20/50 μM: *p* < 0.0001).

Collectively, these cellular experiments confirmed that SCU could dose-dependently alleviate hypoxia-induced HPASMCs overproliferation, reverse abnormal phenotypic transformation and inhibit the secretion of pro-inflammatory cytokines.

### 2.7. Rescue Experiments

To further investigate the cellular protective effects of SCU under hypoxic conditions, EdU incorporation and flow cytometry assays were performed to evaluate cell proliferation and pyroptosis-related changes, respectively.

**Figure 6:**
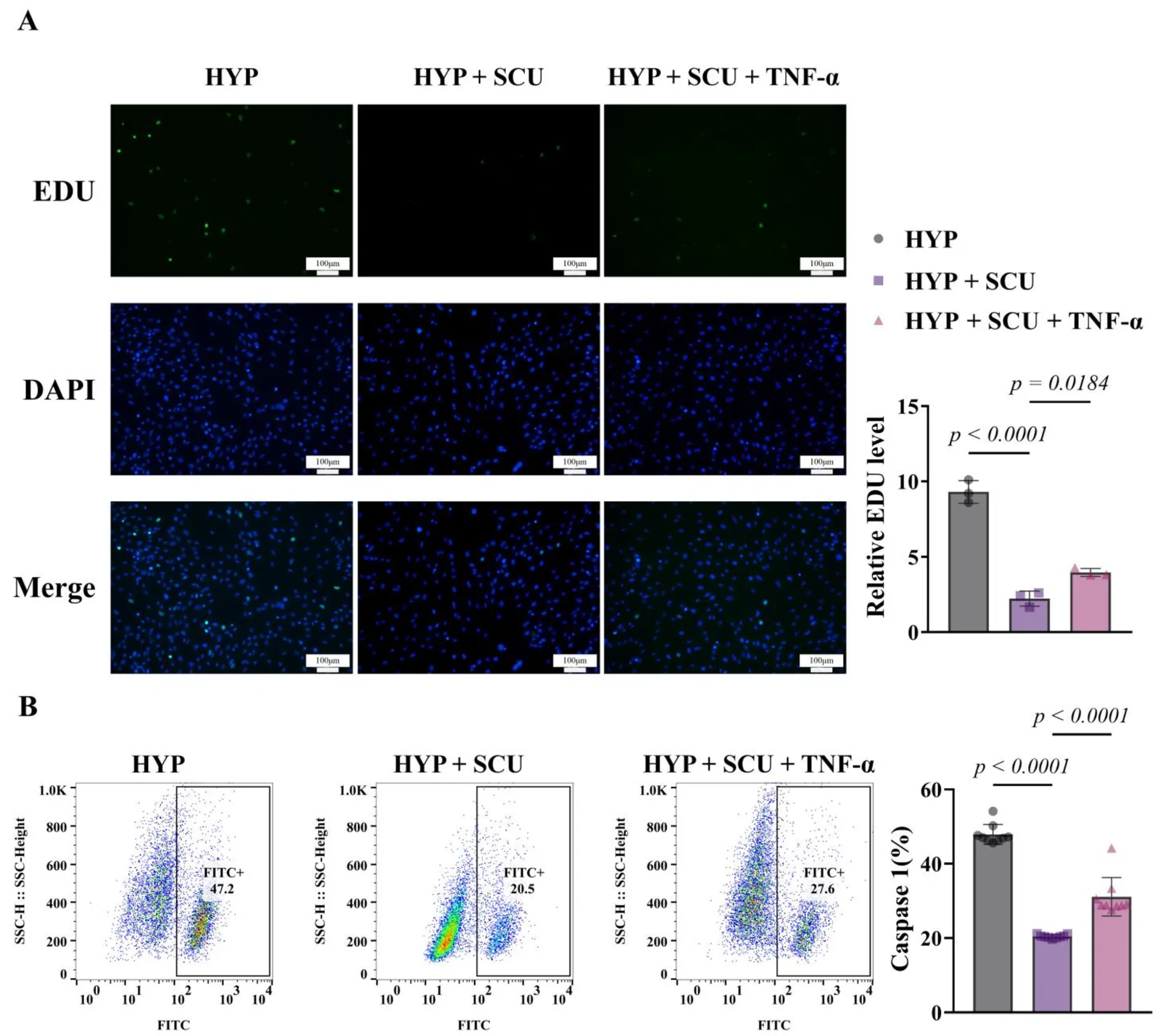
SCU attenuates hypoxia-induced cell proliferation and pyroptosis, whereas TNF-α stimulation partially reverses these effects. (**A**) Representative immunofluorescence images of EdU staining showing proliferating cells under different treatment conditions. Quantitative analysis of relative EdU-positive cells was performed for the HYP, HYP + SCU, and HYP + SCU + TNF-α groups. Scale bar = 100 μm. (**B**) Flow cytometric analysis of caspase-1-positive cells under different treatment conditions. Representative flow cytometry plots and quantitative analysis of caspase-1-positive populations are shown. Data are presented as mean ± SD. *p*-values represent intergroup statistical differences. HYP: hypoxia treatment; SCU: scutellarin; TNF-α: tumor necrosis factor-alpha (NF-κB activator).

As shown in Figure 6A, hypoxia exposure markedly increased the number of EdU-positive cells compared with the levels in the control condition, indicating enhanced proliferative activity under hypoxic stress. SCU treatment significantly reduced the proportion of EdU-positive cells (*p* < 0.0001), suggesting that SCU effectively attenuated hypoxia-induced abnormal cell proliferation. However, subsequent stimulation with TNF-α partially restored the proliferative phenotype, resulting in a significant increase in EdU incorporation compared with the levels in the HYP + SCU group (*p* = 0.0184).

Flow cytometric analysis further demonstrated that hypoxia significantly increased caspase-1-positive cells (*p* < 0.0001), indicating enhanced inflammatory pyroptotic activation (Figure 6B). SCU administration markedly reduced caspase-1 positivity (*p* < 0.0001), suggesting inhibition of hypoxia-induced pyroptosis. Conversely, TNF-α stimulation partially abolished the inhibitory effect of SCU, leading to an increased proportion of caspase-1-positive cells compared with the levels in the SCU-treated group (*p* < 0.0001).

Collectively, these findings indicate that SCU protects cells against hypoxia-induced pathological proliferation and pyroptotic activation, while inflammatory stimulation mediated by TNF-α partially counteracts the beneficial effects of SCU.

### 2.8. Regulatory Effects of SCU Combined with BOS on Hypoxia-Induced Proliferation and Pyroptosis in HPASMCs

This study further compared the protective effects of SCU and the clinically approved PAH-targeted drug bosentan (BOS) on hypoxic human HPASMCs and explored their potential synergistic effects. Five groups were established in this experiment: normoxic control (Ctrl), hypoxia (HYP), HYP + SCU, HYP + BOS, and HYP + SCU + BOS combination groups.

**Figure 7:**
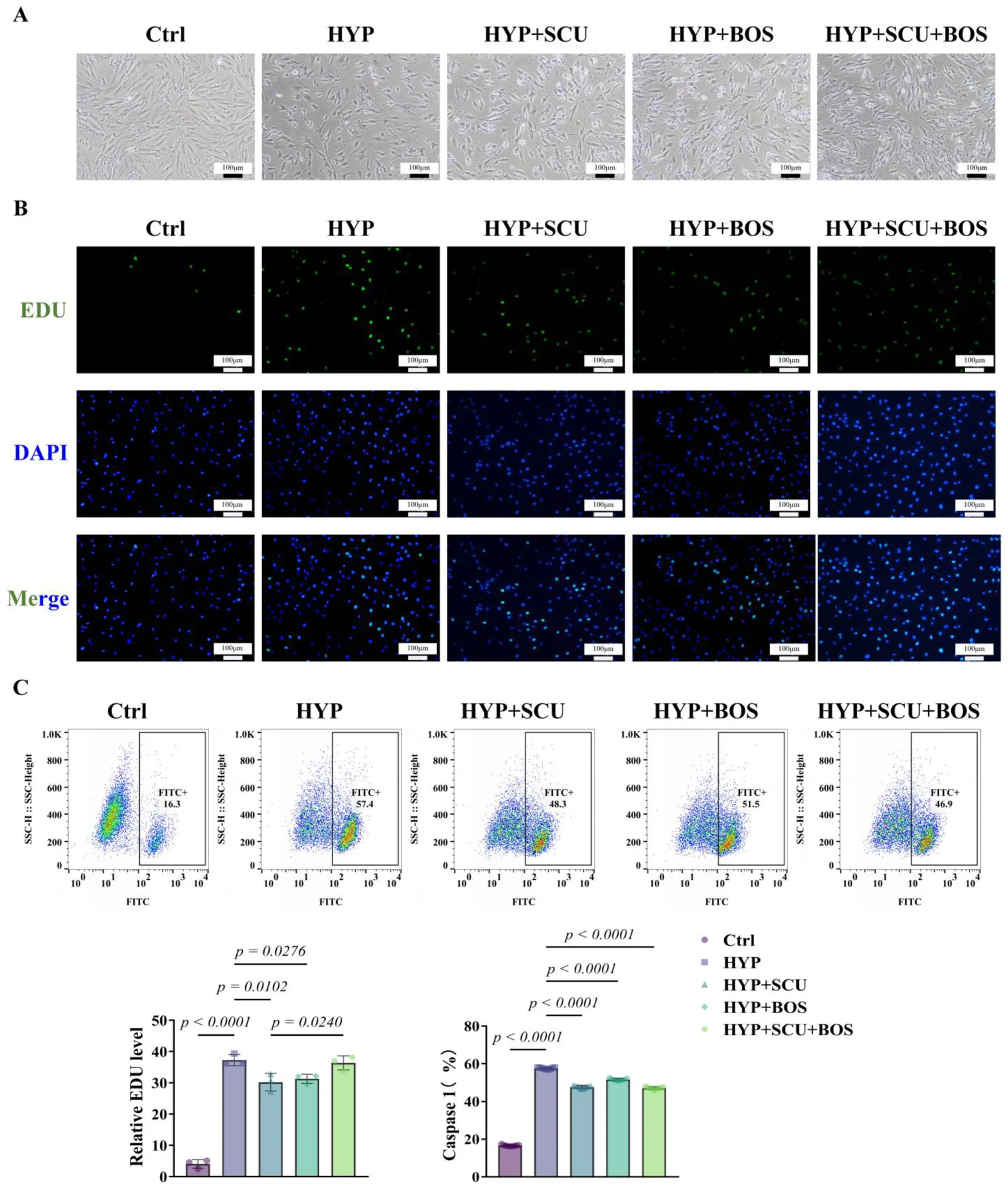
Comparative effects of SCU and bosentan on hypoxia-induced proliferation and pyroptosis of HPASMCs. (**A**) Representative bright-field images showing the morphological changes in HPASMCs in each group; scale bar = 100 μm. (**B**) EdU immunofluorescence staining for proliferative HPASMCs. Green signals represent EdU positive proliferating cells, and blue signals (DAPI) indicate cell nuclei; merged images are presented in the bottom panel; scale bar = 100 μm. (**C**) Flow cytometry scatter plots. Data are presented as mean ± SD. *p*-values represent intergroup statistical differences. Ctrl: normoxic control group; HYP: hypoxia group; SCU: scutellarin; BOS: bosentan; EdU: 5-ethynyl-2′-deoxyuridine; DAPI: 4′,6-diamidino-2-phenylindole.

Microscopic observation (Figure 7A) showed that hypoxia induced hypertrophic morphology and disordered arrangement in HPASMCs. Both SCU and BOS monotherapy partially restored the normal spindle shape of hypoxic cells, while combined treatment exerted a more prominent improvement in abnormal cellular morphology.

EdU staining results (Figure 7B) revealed a significantly increased proportion of proliferative cells in the HYP group compared with the levels in the Ctrl group (*p* < 0.0001). Single treatment with SCU or BOS notably inhibited hypoxia-induced excessive proliferation (HYP vs. HYP + SCU: *p* = 0.0102; HYP vs. HYP + BOS: *p* = 0.0276), and their combination further suppressed cell proliferation (*p* = 0.0240 vs. HYP group).

Flow cytometry analysis of active caspase-1-positive cells was performed to evaluate cellular pyroptosis (Figure 7C). Hypoxia markedly elevated the pyroptotic cell ratio (*p* < 0.0001), whereas SCU or BOS monotherapy strongly reversed this phenotype (both *p* < 0.0001). The co-treatment group showed the lowest pyroptosis level, with a highly significant difference from that of the HYP group (*p* < 0.0001).

In summary, SCU effectively attenuates hypoxia-induced overproliferation and pyroptosis in HPASMCs, with a therapeutic efficacy comparable to that of the first-line PAH drug bosentan. No obvious synergistic effect was observed after combined administration, indicating potential overlap in their regulatory signaling pathways.

## 3. Discussion

Excessive inflammatory activation and aberrant cell death are central pathological mechanisms in the development and progression of PAH [16,17], among which inflammation and pyroptosis mediated by the NF-κB/NLRP3 signaling axis play a pivotal role in disease pathogenesis [18,19,20]. In the present study, SCU, a flavonoid bioactive compound derived from Erigeron breviscapus, was demonstrated to significantly ameliorate pulmonary vascular remodeling and right ventricular dysfunction in PAH model rats. Mechanistically, SCU inhibited the NF-κB/NLRP3 signaling pathway, thereby suppressing downstream inflammatory cascades and GSDMD-mediated pyroptosis, ultimately exerting a protective effect on pulmonary vasculature.

Previous studies have shown that SCU possesses multiple cardiovascular pharmacological activities, including anti-inflammatory, anti-apoptotic, anti-fibrotic, and endothelial-protective effects [21,22]. However, its role in PAH and the underlying molecular mechanisms of its effects remain incompletely understood. In this study, both in vivo and in vitro experiments systematically demonstrated the therapeutic efficacy of SCU in PAH. In animal models, SCU significantly improved right ventricular functional parameters, resulting in an increased PAT/PET ratio, reduced RVAW, and improved TAPSE. Moreover, it alleviated pulmonary arteriolar wall thickening, lumen narrowing, and perivascular collagen deposition, thereby suppressing pathological vascular remodeling and fibrosis. In vitro, SCU healed hypoxia-induced injury in HPASMCs and inhibited abnormal cell proliferation. These consistent in vivo and in vitro findings collectively indicate that SCU is a promising therapeutic candidate for PAH.

To date, preclinical studies investigating the protective effects of SCU against PAH-associated vascular injury remain limited. Previous studies have demonstrated that SCU can alleviate monocrotaline (MCT)-induced PAH, primarily through the inhibition of oxidative stress and preservation of endothelial function. Unlike the MCT model used in earlier studies, the present study employed a SuHx-induced rat model, which more closely replicates the key pathological features of human PAH, including severe pulmonary vascular remodeling, perivascular fibrosis, and right ventricular dysfunction. Importantly, the novelty of this study lies in further identifying NF-κB/NLRP3-mediated inflammation and pyroptosis as critical downstream mechanisms underlying the protective effects of SCU. Consistent with previous cardiovascular studies of SCU, our findings further demonstrate that SCU exerts anti-inflammatory and anti-remodeling effects in pulmonary tissue.

Pyroptosis is a pro-inflammatory form of programmed cell death characterized by membrane rupture and massive release of inflammatory mediators, thereby exacerbating pulmonary vascular injury and remodeling and contributing to PAH progression [23,24,25]. During PAH development, hypoxic and inflammatory stimuli induce excessive pyroptosis in pulmonary vascular cells, leading to the release of IL-1β and IL-18, which amplify inflammatory responses and promote abnormal proliferation of pulmonary artery smooth muscle cells, thereby forming a vicious “inflammation-pyroptosis-vascular remodeling” cycle [26,27]. In this study, expression levels of NLRP3, cleaved caspase-1, GSDMD-N, IL-1β, and IL-18 were significantly increased in lung tissues of PAH rats, indicating aberrant activation of the NLRP3 inflammasome. SCU markedly downregulated both the mRNA and protein levels of these pyroptosis-related molecules, inhibited caspase-1/GSDMD-mediated pyroptosis, and alleviated structural vascular damage.

NF-κB acts upstream of NLRP3 inflammasome activation and initiates downstream pyroptotic signaling [28,29]. Upon activation, NLRP3 recruits ASC and pro-caspase-1 to assemble a functional inflammasome complex. Activated caspase-1 cleaves GSDMD and pro-IL-1β/IL-18, leading to the release of mature IL-1β and IL-18 and triggering pyroptosis-associated inflammatory amplification. Previous studies have mainly focused on downstream inhibition strategies, such as specific NLRP3 blockade using MCC950 to alleviate PAH injury [30,31].

In this study, molecular docking results demonstrated that SCU stably binds to both p50 and p65 subunits of NF-κB. Given that p50 and p65 share a conserved Rel homology domain [32], they form a structurally similar binding pocket. We therefore propose that SCU interacts with conserved amino acid residues within these subunits via hydrogen bonding, occupying the active site. This dual-targeting mechanism may interfere with multiple steps of NF-κB activation via the disruption of p50-mediated DNA binding, inhibition of p65 recruitment of co-activators, and impairment of p50/p65 heterodimer formation and nuclear translocation, ultimately suppressing the transcription of pro-inflammatory and pro-pyroptotic genes and reducing pulmonary inflammation and vascular remodeling in PAH.

Molecular-docking simulations for the JUN-DNA complex predict contacts between SCU and DNA nucleotides rather than the direct physical binding of SCU to the JUN protein. This computational hypothesis requires further biochemical experimental validation.

Notably, we observed a discrepancy in IκBα expression between mRNA and protein levels: although significant intergroup differences in IκBα mRNA levels were detected, the total protein expression did not change markedly. This phenomenon may reflect the intrinsic dynamic regulatory mechanism of the NF-κB pathway rather than a simple expression artifact. Recent transcriptomic studies have demonstrated that hypoxia specifically activates the IKK-NF-κB signaling axis. Activated p65 can directly bind to the NFKBIA promoter and stimulate IκBα transcription, and this process relies on an intact IKK signaling pathway [33], which is consistent with the classic NF-κB negative-feedback regulatory model [34].

On one hand, under persistent hypoxic stimulation, activated NF-κB promotes NFKBIA transcription to compensate for signal activation, and on the other hand, it induces continuous phosphorylation, ubiquitination, and proteasomal degradation of IκBα. Owing to the high turnover rate of newly synthesized IκBα protein, elevated transcription may not be sufficient to counterbalance ongoing protein degradation, resulting in increased IκBα mRNA, accompanied by relatively stable total protein levels [35]. Therefore, the discordance between IκBα mRNA and protein expression may represent a dynamic balance formed between transcriptional regulation and protein homeostasis during NF-κB signal activation. Furthermore, although the increased p-IκBα level under SuHx stimulation supports activation of the canonical IKK/IκBα/NF-κB pathway, we cannot exclude the involvement of alternative NF-κB regulatory mechanisms or parallel signaling pathways, which will require further investigation.

Inflammation, extracellular matrix deposition, and mesenchymal activation are central drivers of vascular fibrosis in PAH. MMP-9 and vimentin are key markers of vascular remodeling and mesenchymal activation, directly regulating extracellular matrix remodeling and fibrotic progression [36,37]. In this study, SCU significantly downregulated MMP-9 and vimentin expression, suggesting that it suppresses inflammation and pyroptosis mediated by the NF-κB/NLRP3 axis, thereby indirectly inhibiting vascular remodeling and fibrosis. These multi-target effects further highlight its therapeutic potential in PAH.

Given the complex and multifactorial pathogenesis of PAH, combination therapeutic strategies targeting distinct pathological mechanisms have attracted increasing attention [38]. In the present study, we compared the effects of SCU and bosentan, a clinically approved targeted drug for PAH, both individually and in combination using a hypoxia-induced HPASMC model. Both SCU and bosentan significantly inhibited hypoxia-triggered cell proliferation and pyroptotic responses; however, no statistically significant synergistic effect was observed upon combined treatment. This finding suggests that the therapeutic effects of SCU and bosentan may not be fully additive within this simplified cellular system.

We consider that the HPASMC model only reflects the abnormalities of vascular smooth muscle cells and cannot replicate the complex pathological interactions of PAH, including endothelial dysfunction, immune-cell infiltration, inflammatory microenvironment, hemodynamic alterations, and right ventricular remodeling. In addition, bosentan mainly improves pulmonary vascular tone via endothelin receptor blockade [39], whereas SCU primarily suppresses inflammatory activation, the NF-κB/NLRP3 signaling pathway, and pyroptosis. Therefore, whether the combination of SCU and bosentan can yield additional therapeutic benefits requires further validation in clinically relevant animal models and pharmacological studies.

Of note, the absence of obvious synergistic effects observed in our in vitro HPASMC model does not preclude potential combinatorial benefits upon combined SCU-bosentan treatment in vivo. The simplified hypoxic cell-culture system cannot replicate the full complexity of PAH pathogenesis, including hemodynamic disturbance, right-ventricular remodeling, endothelial injury and immune-cell infiltration. Bosentan mainly improves hemodynamics via endothelin-receptor-mediated pulmonary vasodilation, whereas SCU targets the NF-κB/NLRP3-pyroptosis cascade to suppress inflammation, pyroptosis and fibro-proliferative vascular remodeling. Their divergent mechanisms of action may produce complementary therapeutic effects in intact animal models. Further in vivo pharmacological investigations are therefore warranted to systematically evaluate the therapeutic value of SCU combined with bosentan for PAH.

This study has several limitations. The precise binding sites and interaction kinetics between SCU and the NF-κB/NLRP3 axis remain to be further validated using structural biology approaches and site-directed mutagenesis. In addition, the pharmacokinetic profile, long-term safety, and optimal dosing regimen of SCU have yet to be systematically investigated in large animal models and clinical trials, which are essential to fully support its translational potential. Notably, our in silico ADME prediction indicated that scutellarein (SCU) exhibits relatively poor gastrointestinal absorption, which may substantially limit its oral bioavailability. Low oral bioavailability represents a major pharmacokinetic barrier hindering the clinical translation of SCU. Although SCU has demonstrated promising anti-inflammatory and anti-pyroptotic activities in preclinical animal models, insufficient intestinal absorption following oral administration may result in inadequate drug exposure at pulmonary target tissues. Therefore, before advancing SCU toward large-animal studies and clinical trials, future translational investigations should consider formulation-based optimization strategies, such as nanoparticle-based delivery systems or prodrug modification, to improve systemic bioavailability and enhance drug accumulation at target sites. Furthermore, additional studies are warranted to determine whether combination therapy with SCU and bosentan provides superior therapeutic efficacy compared with the results for either monotherapy.

Moreover, it should be noted that SCU was administered synchronously with SU5416 injection and hypoxia initiation in the current animal experiments, corresponding to a preventive intervention paradigm. This experimental setting differs from real-world clinical practice, where PAH patients are generally diagnosed and commence treatment after pulmonary vascular remodeling has already been established. Therefore, the protective effect observed under this preventive regimen cannot be directly extrapolated to the clinical therapeutic scenario. Future in vivo studies employing therapeutic drug administration (treatment initiated after stable PAH pathological phenotypes are fully developed) are warranted to further evaluate the clinically relevant efficacy of SCU.

Finally, it should also be noted that network pharmacology, molecular docking and molecular dynamics simulation are computational prediction tools with notable intrinsic limitations. Network-pharmacology analysis is primarily used to generate mechanistic hypotheses instead of to provide direct experimental proof. Bioinformatic output cannot fully replicate complex intracellular contexts, including dynamic protein-protein crosstalk, post-translational modifications, subcellular localization, and real-time drug-target engagement under physiological or pathological conditions. Likewise, molecular-docking simulations represent only in silico predictions of potential ligand-target interactions. The calculated binding free energies and predicted binding poses are simulated outcomes and are not equivalent to real physical binding inside biological systems. Reliable confirmation of SCU-target interactions will require follow-up biochemical assays such as surface plasmon resonance (SPR), microscale thermophoresis (MST), and site-directed mutagenesis experiments. Therefore, all computational-derived mechanistic hypotheses in this study await further wet-laboratory experimental validation.

## 4. Materials and Methods

### 4.1. SCU Target Screening and Drug Similarity Prediction

To predict the potential targets of SCU, we retrieved information from multiple public databases, including the Traditional Chinese Medicine Systems Pharmacology Database and Analysis Platform (TCMSP, https://old.tcmsp-e.com/tcmsp.php, accessed on 19 June 2020), the Search Tool for Interactions of Chemicals (STITCH, http://stitch.embl.de/), and the DrugBank Drug and Drug Target Database (DrugBank, http://www.drugbank.ca). The collected targets were subjected to rigorous aggregation and deduplication, followed by uniform conversion to official gene symbols using the UniProt and NCBI platforms to ensure data standardization and consistency. Subsequently, the Simplified Molecular-Input Line-Entry System (SMILES) notation of SCU was obtained from the PubChem database. The online platform SwissADME (http://www.swissadme.ch/) was employed to predict and evaluate the physicochemical properties, pharmacokinetic (absorption, distribution, metabolism, and excretion, ADME) characteristics, and drug-likeness of SCU, for a preliminary assessment of its druggability potential.

### 4.2. Identification of Targets Associated with PAH

To identify disease-relevant targets of PAH, we performed targeted searches against multiple disease-gene databases, including GeneCards (https://www.genecards.org/), OMIM (https://omim.org/), and DisGeNET (https://www.disgenet.org/), using the keywords Pulmonary Arterial Hypertension and Pulmonary Hypertension. The retrieved gene entries were downloaded, integrated, and subjected to strict deduplication to construct a curated PAH disease target dataset.

### 4.3. Identification of Core Compound-Disease Targets and Network Construction

Based on the aforementioned screening results, the online tool Draw Venn Diagram (https://bioinformatics.psb.ugent.be/webtools/Venn/, accessed on 19 June 2020) was applied to perform intersection analysis between potential targets of SCU and PAH-related disease targets. The overlapping genes obtained were defined as the core therapeutic targets of SCU against PAH. Furthermore, the compound-core target interaction network was established and visualized using Cytoscape 3.10.3 software, aiming to systematically elucidate the multi-target regulatory mechanism underlying the therapeutic effects of SCU in PAH treatment.

### 4.4. Construction of PPI Network and Screening of Core Hub Targets

To further explore the interactive relationships among the core therapeutic targets, the identified core targets were imported into the STRING database (Version 12.0, https://cn.string-db.org/) for PPI network analysis. In this analysis, the species was restricted to “Homo sapiens”, and the minimum required interaction score was set to a high-confidence threshold of 0.900. Unconnected isolated nodes were subsequently excluded to optimize the network. The resultant PPI data were downloaded in TSV format and imported into Cytoscape 3.10.3 software. The built-in Network Analyzer plugin was utilized to calculate pivotal topological parameters, including degree centrality (DC), betweenness centrality (BC), and closeness centrality (CC). Node topological filtering was performed through a two-step median threshold strategy: First, nodes with DC, BC, and CC values exceeding or equal to the network-wide medians (DC ≥ Median, BC ≥ Median, CC ≥ Median) were retained to establish a candidate target network; second, a second-round calculation was conducted on this sub-network using the same criteria to identify the ultimate core hub targets.

### 4.5. Functional Enrichment Analysis and TFs Identification

To systematically characterize the biological functions of core therapeutic targets, Gene Ontology (GO) enrichment analysis, including biological process (BP), cellular component (CC), and molecular function (MF) subcategories, was performed, alongside Kyoto Encyclopedia of Genes and Genomes (KEGG) pathway enrichment analysis, using the clusterProfiler package in R. Concurrently, Reactome pathway annotation was conducted using the ReactomePA package. A *p*-value < 0.05 was set as the threshold for statistical significance in all enrichment analyses. For clear visualization, the top 5, top 10, and top 20 terms with the lowest *p*-values were selected from the GO, KEGG, and Reactome enrichment results, respectively. Additionally, to explore the transcriptional regulatory mechanisms of these core targets, TF identification for the SCU-PAH core target set was performed using the relevant module of the R package TCMNP.

### 4.6. Molecular Docking Validation

To evaluate the binding affinity between SCU and core hub targets screened via network pharmacology, the two-dimensional (2D) structure of SCU was retrieved from the PubChem database. Geometric optimization and energy minimization were subsequently performed using ChemBio3D Ultra (version 14.0.0.117). The optimized structure was converted into the 3D PDBQT format as the ligand for subsequent docking with AutoDock Vina (version 1.2.7). Meanwhile, the three-dimensional crystal structures of core hub targets with corresponding PDB IDs were downloaded from the Protein Data Bank (PDB, https://www.rcsb.org/). All receptor proteins were pretreated with AutoDock Tools (version 1.5.7), including the removal of redundant water molecules and non-essential small molecules, as well as hydrogen addition. Molecular docking calculations were carried out using AutoDock Vina 1.1.2. The optimal docking conformation was selected based on the minimum binding energy, and three-dimensional visual analysis of the docking results was accomplished using PyMOL (version 3.0.3).

### 4.7. Molecular Dynamics Simulation and Analysis

The initial structure of the p50-scutellarein complex was obtained from the most favorable docking pose generated by AutoDock Vina using the NF-κB p50 structure (rep_nfkb.pdb, residues 43–353). The protein was parameterized using the AMBER14SB force field, while ligand parameters were generated using the GAFF2 force field with AM1-BCC atomic charges. The complex was solvated in an explicit TIP3P water box with a 10 Å buffer distance, and counterions were added to neutralize the system, followed by the addition of 0.15 M NaCl to mimic physiological ionic strength. Energy minimization was initially performed to remove unfavorable contacts, followed by NVT equilibration at 300 K for 100 ps and subsequent NPT production simulation. A 100 ns molecular dynamics trajectory was generated, and the equilibrium production phase from 28.1 to 99.9 ns was selected for further analysis, resulting in 719 trajectory frames collected at 100 ps intervals. All structural analyses were performed under the minimum-image convention (MIC). Periodic boundary condition (PBC) corrections and minimum-image translations were applied to the ligand coordinates. Protein Cα atoms were aligned to the reference structure at 28.1 ns to eliminate overall translational and rotational motions. The root-mean-square deviation (RMSD), radius of gyration (Rg), solvent-accessible surface area (SASA), root-mean-square fluctuation (RMSF), intermolecular distances, and binding-pocket volume were calculated using MDTraj. Hydrogen bonds were identified based on a donor-acceptor distance cutoff of <3.5 Å and a donor-hydrogen-acceptor angle > 150°. Protein-ligand interaction energies were calculated using AMBER14SB and GAFF2/AM1-BCC parameters, including Coulombic and Lennard-Jones interactions, with a cutoff distance of 1.2 nm. These interaction energies represent only nonbonded interaction contributions and were used for comparative analysis rather than absolute binding free-energy estimation. DCCM analysis and principal component analysis (PCA) were performed based on protein Cα coordinates. The free-energy landscape (FEL) was constructed using Boltzmann inversion based on a 14 × 14 histogram projected onto the first two principal components (PC1 and PC2), with energy values expressed in kJ/mol.

### 4.8. Animals

A total of 24 male Sprague Dawley (SD) rats, 6–8 weeks old, weighing 200–250 g, were purchased from the Laboratory Animal Center of Kunming Medical University. All animals were housed in a SPF barrier environment under standardized conditions, i.e., a constant temperature of 22 ± 2 °C, relative humidity of 55 ± 10%, and a 12 h light/dark cycle, with free access to standard diet and sterile water. All rats were acclimatized for one week prior to formal experimentation. For the in vivo animal experiments in this study, *n* = 6 biological replicates were set for each group, with three technical replicates.

All animal experimental protocols were reviewed and approved by the Ethics Committee of Kunming Medical University (Approval No.: kmmu20242004). All animal operations were strictly implemented in accordance with the Guide for the Care and Use of Laboratory Animals (NIH Publication No. 85-23, revised 2011).

### 4.9. Reagents and PAH Animal Model Establishment

Rats were randomly divided into four groups with six rats per group (*n* = 6). Normal control group (Ctrl): Rats received a single subcutaneous injection of an equal volume of SU5416 blank vehicle to match the injection procedure of model groups, were raised under normoxic conditions (21% O_2_), and were intragastrically administrated an equal volume of ethanol vehicle daily for 3 consecutive weeks. PAH model group (SuHx): Rats were subcutaneously injected with SU5416 solution and exposed to 3 weeks of intermittent hypoxia. The hypoxic chamber (Ox-100, Tongwei Technology, Shanghai, China) was used to establish intermittent-hypoxia conditions. The oxygen concentration was maintained at 10 ± 0.5% O_2_ by continuous nitrogen gas displacement, following a 12 h hypoxia/12 h normoxia alternating cycle for 3 consecutive weeks. During normoxia phases, the chamber was restored to normal room-air oxygen levels (~21% O_2_). Ambient temperature and relative humidity inside the chamber were kept consistent with the general animal housing environment (22 ± 2 °C, 55 ± 10% humidity). An equal volume of ethanol vehicle was given by daily gavage throughout modeling to establish stable PAH lesions.

After 3 weeks of modeling, all rats first underwent echocardiographic assessment under light isoflurane inhalation anesthesia, as described in Section 4.10. Upon completion of cardiac function detection, rats were deeply anesthetized via intraperitoneal injection of 2% sodium pentobarbital (Merck Chemicals (Shanghai) Co., Ltd., Shanghai, China, 11715-500G), with sustained low-dose isoflurane inhalation applied to maintain stable anesthesia throughout tissue collection. Subsequently, lung tissues were perfused and fixed in situ, and cardiac and pulmonary specimens were harvested for subsequent morphological and molecular biological detection.

Both semaxanib (SU5416; MedChemExpress, Shanghai, China, HY-10374) and SCU (Beijing Solarbio Science & Technology Co., Ltd., Beijing, China, ISO590) had a purity ≥ 98%. All compounds were fully dissolved in dimethyl sulfoxide (DMSO). The SU5416 stock solution (22.5 mg/mL) was prepared with mixed vehicle (DMSO: PEG300:Tween-80:normal saline = 0.1:0.8:0.1:1.0). The SCU working solution was configured at 1.5 mg/mL, initially dissolved in DMSO and further diluted with ethanol solution.

### 4.10. Echocardiography Assessment

At the experimental endpoint, rats were lightly anesthetized with isoflurane inhalation (induction flow rate: 0.8–1.0 L/min; maintenance flow rate: 0.2–0.3 L/min), and echocardiographic detection was immediately performed. Stable ultrasonic images were acquired using a color Doppler ultrasound diagnostic system (M9T, Shenzhen Mindray Bio-Medical Electronics Co., Ltd., Shenzhen, China). Key indicators were measured, i.e., PAT/PET ratio, RVAW, and TAPSE. The PAT/PET ratio was further calculated to comprehensively evaluate pulmonary hemodynamics [40,41] and right ventricular cardiac function.

### 4.11. HE Staining

Lung tissues isolated from each group were fixed in 4% paraformaldehyde (Guangfu, Tianjin, China, CNAB035-Q) overnight. After gradient dehydration with ethanol (Chengdu Kelong Chemical Co., Ltd., Chengdu, China, GB/T678-2002) and transparency achieved with xylene (Xilong Scientific Co., Ltd., Shantou, China, 33535), tissues were embedded in paraffin and cut into 5 μm thick continuous sections for HE staining.

Paraffin sections were baked in a constant-temperature incubator (303-2, Dongguan Yihui Optoelectronics Technology Co., Ltd., Dongguan, China) at 64 °C for 1 h, followed by xylene dewaxing and gradient ethanol rehydration. Sections were then stained with hematoxylin (Wuhan Servicebio Technology Co., Ltd., Wuhan, China, G1004-100 mL) and eosin (Solarbio), dehydrated with anhydrous ethanol, cleared with xylene, and sealed with neutral balsam (Servicebio, WG10004160). Representative visual fields were randomly selected and observed under an optical microscope. Histological images were captured using SlideViewer software (3DHISTECH, version 2.6).

Pulmonary arterioles with diameters ranging from 50 μm to 100 μm were selected for morphometric quantitative analysis. Vascular remodeling severity was assessed by calculating the percentage of WT% and WA%.

### 4.12. Masson’s Trichrome Staining

Pulmonary paraffin sections were stained using a commercial Masson’s trichrome staining kit (G1340, Solarbio). Briefly, sections were immersed in Masson solution A at room temperature overnight and incubated in a 65 °C oven for 30 min. After thorough rinsing, sections were dyed with the mixed solution of Masson B and Masson C, differentiated with 1% hydrochloric acid-alcohol (concentrated hydrochloric acid:absolute ethanol = 1:100), and sequentially incubated in Masson D, Masson E and Masson F solutions. Following differentiation with dilute glacial acetic acid solution, sections were dehydrated with anhydrous ethanol, cleared with xylene (Xilong Scientific, 33535), and mounted with neutral balsam (Servicebio, WG10004160). Random microscopic fields were photographed for subsequent analysis.

Image analysis was performed via SlideViewer and other professional imaging software to quantify the percentage of collagen deposition area (Area%) so as to evaluate the fibrosis degree in lung parenchyma and perivascular tissues.

### 4.13. Western Blot

Approximately 50 mg of frozen left lung tissues was lysed in pre-cooled RIPA lysis buffer (Servicebio, G2002-30 mL) supplemented with 1% protease and phosphatase inhibitor cocktail. Tissue homogenization was conducted using a high-speed homogenizer (30 Hz, 30 s, repeated three times). The tissue lysate was centrifuged at 12,000× *g* for 15 min at 4 °C, and the supernatant was collected for subsequent protein quantification.

Total protein concentration was precisely determined using a BCA protein assay kit (Beyotime Biotechnology, Shanghai, China, P0009). Equal amounts of protein samples were mixed with 5×SDS loading buffer and denatured at 95 °C for 10 min. Protein samples were separated by 10% SDS-PAGE electrophoresis (80 V for stacking gel, 120 V for separating gel) and electrotransferred onto PVDF membranes (EMD Millipore Corporation, Burlington, MA, USA, K2MA8350E).

Membranes were blocked with 5% skimmed milk powder at room temperature for 1 h and then incubated with primary antibodies (detailed in Table S1, including manufacturer information and dilution ratios) diluted in 5% BSA solution at 4 °C overnight. After three washes with TBST buffer containing 0.1% Tween-20 (10 min per wash), membranes were incubated with HRP-conjugated secondary antibodies at room temperature for 1 h.

Protein bands were visualized using ECL chemiluminescence reagents (Affinity Biosciences Ltd., Liyang, China, KF8001), and signal images were captured by the ChemiDoc XRS + imaging system. The gray value of each target band was quantitatively analyzed with ImageJ software (version 1.8.0.345). β-actin was used as the internal reference for normalization, and the relative protein expression levels were calculated accordingly.

The relative protein expression levels were quantified and normalized to β-actin as the internal reference.

### 4.14. RNA Extraction and Quantitative RT-qPCR

Total RNA was isolated from lung tissues and cultured cells using Trizol reagent (Vazyme, Nanjing, China, R401-01). The concentration of total RNA was adjusted to 100 ng/μL, and RNA purity was evaluated via the A260/280 absorbance ratio using a NanoDrop spectrophotometer. Complementary DNA (cDNA) was synthesized with a commercial reverse transcription kit (Beijing Codonx Biotechnology Co., Ltd., Beijing, China, KR0501). Relative mRNA expression levels were quantified by RT-qPCR using the SYBR Premix EX Taq kit (Servicebio, G3326-05) on an Applied Biosystems 7500 Fast Real-Time PCR system. The thermal cycling protocol was set as follows: initial denaturation at 95 °C for 30 s, followed by 40 cycles of 95 °C for 5 s and combined annealing-extension at 60 °C for 30 s. GAPDH was employed as the internal reference gene, and the relative transcriptional levels of target genes were calculated using the 2^−ΔΔCt^ method. All primer sequences used in this study were synthesized by Sangon Biotech (Shanghai, China). Detailed primer information for lung tissues and HPASMCs is presented in Table S2 and Table S3, respectively.

### 4.15. Cell Culture

HPASMCs were routinely cultured in complete medium consisting of 89% F12 special medium (Dalian Meilun Biotechnology Co., Ltd., Dalian, China, MA213), 10% fetal bovine serum (FBS), and 1% penicillin-streptomycin double antibiotics. Cells were maintained under standard culture conditions and passaged at an appropriate confluence. Subsequent interventions were designed as follows: normal control group (Ctrl), 24 h hypoxia stimulation group (HYP), vehicle control group (Vehicle), and hypoxic groups co-treated with gradient concentrations of SCU (5, 10, 20 and 50 μM) or bosentan as the positive drug for 24 h. Bosentan (MedChemExpress, Shanghai, China, HY-A0013, purity: 99.94%) was dissolved in DMSO to prepare stock solutions for in vitro cell treatments. For all cell-based experiments in this study, we used *n* = 3 biological replicates per group, and each biological replicate included three technical replicates.

### 4.16. Cell Viability Assay (CCK-8)

HPASMCs from each treatment group were digested, centrifuged and counted. Cells were seeded into 96-well plates at a density of 3000–7000 cells per well with a final volume of 100 μL, and three replicate wells were set for each group. Cells were incubated at 37 °C with 5% CO_2_ and 90% humidity for 0, 12, 24, 48 and 72 h. Afterwards, CCK-8 working solution (Elabscience Biotechnology Co., Ltd., Wuhan, China, E-CK-A362) was added to each well, and the plates were incubated for another 2 h at 37 °C. The absorbance value at 450 nm was finally measured to reflect cell viability.

### 4.17. Cell Proliferation Assay

HPASMCs were harvested, resuspended and seeded into 96-well plates at 3000–7000 cells/well with three biological replicates. According to the manufacturer’s instructions for the EdU-488 cell proliferation detection kit (Beyotime, C0071S), cells were incubated with 10 μM EdU working solution for 2 h. Subsequently, cells were fixed with 4% paraformaldehyde (Guangdong Guanghua Sci-Tech, Shantou, China, BL539A) for 15 min at room temperature and permeabilized with 0.3% Triton X-100 in PBS (Labgic Technology Co., Ltd. (Biosharp brand), Beijing, China, BL302A) for 15 min. After incubation with Click reaction solution for 30 min in the dark at room temperature, cell nuclei were counterstained with Hoechst 33342. Fluorescence signals were detected and photographed; Hoechst 33342 emitted blue fluorescence with a maximum excitation wavelength of 346 nm and a maximum emission wavelength of 460 nm.

### 4.18. Flow Cytometric Quantification of Caspase-1-Positive Pyroptotic HPASMCs

HPASMCs with different treatments were digested with trypsin (Biosharp, Beijing, China, BL512A), and the cell suspension was transferred to EP tubes. Cells were harvested by centrifugation at 300× *g* for 5 min, and the supernatant was discarded. According to the manufacturer’s instructions for the Caspase 1 Activity Detection Substrate for Flow Cytometry kit (Elabscience, Biotechnology Co., Ltd., Wuhan, China, E-CK-A481), cell pellets were resuspended in 400 μL PBS, followed by the addition of 0.4 μL Caspase 1 Substrates (Green). The mixture was gently pipetted to homogenize the solution, yielding a final substrate concentration of 1 μM, and incubated at 37 °C in the dark for 30 min. After incubation, 1 mL PBS was added to resuspend cells, and centrifugation was performed at 250× *g* for 5 min to remove the supernatant. The cell pellets were reconstituted with 500 μL PBS for flow cytometric detection. A total of 10,000 cells were collected via the BD FACSCalibur flow cytometer, and the proportion of caspase-1-positive pyroptotic cells was analyzed using FlowJo software (version 10.8.1).

### 4.19. Statistical Analysis

All quantitative data were processed using SPSS 20.0, and graphical visualization was performed using GraphPad Prism 8.0. Experimental results were expressed as mean ± standard deviation (Mean ± SD). Prior to one-way analysis of variance (one-way ANOVA), the normality of data distribution and homogeneity of variance were verified using the Shapiro-Wilk test and Levene’s test, respectively. One-way ANOVA was adopted for multi-group comparison, followed by Tukey’s post hoc test for multiple pairwise comparisons. A two-tailed *p* < 0.05 was defined as the threshold of statistical significance.

## 5. Conclusions

SCU significantly ameliorates pulmonary vascular remodeling and right ventricular dysfunction in PAH rats. Mechanistically, this effect is mediated via inhibition of the NF-κB/NLRP3 pathway, blocking downstream inflammatory responses and GSDMD-dependent pyroptosis, thereby reducing inflammatory injury and fibrotic remodeling. These findings establish SCU as a promising natural agent for targeting inflammation and pyroptosis in PAH.

Nevertheless, intrinsic pharmacokinetic limitations, including relatively poor oral bioavailability, still restrict its direct clinical application. Future work should focus on formulation optimization, large-animal pharmacological evaluation, and safety assessment to overcome these translational obstacles. Collectively, our study provides a solid pre-clinical foundation supporting the further translational development of SCU for pulmonary vascular disease intervention.

## Figures and Tables

**Table 1:** Detailed hydrogen bond interactions between SCU and core target proteins.

Ligand Name	Protein Complex (PDB ID)	ΔG (kcal/mol)	Protein/DNA Residue	Residue Category	Interaction Type	Hydrogen Bond Length (Å)
SCU	NFKB1 (PDB ID: 1SVC)	−7.1	PRO-29	Amino acid (PRO)	Hydrogen Bond	1.8
SCU	IL18 (PDB ID: 1J0S)	−7.1	ARG-27	Amino acid (ARG)	Hydrogen Bond	2.4, 2.6
SCU	JUN (PDB ID: 1JNM)	−9.4	DG-308; DC-213	Deoxyribonucleotide (dG, dC)	Hydrogen Bond	2.7; 2.4, 2.8, 2.4
SCU	RELA (PDB ID: 7LEU)	−6.4	SER-483	Amino acid (SER)	Hydrogen Bond	3.2
SCU	TNF (PDB ID: 1A8M)	−4.2	GLN-88	Amino acid (GLN)	Hydrogen Bond	2.0

Note: Data in the column “Hydrogen Bond Length (Å)” represent the length of each hydrogen bond. ΔG = binding free energy (kcal/mol). For the JUN complex (PDB ID: 1JNM), SCU forms hydrogen bonds with the DNA double-strand nucleotides within the protein-DNA composite crystal structure, not with JUN amino-acid residues. This represents an in silico-only prediction and requires biochemical experimental validation.

## Data Availability

The original data presented in the study are openly available in TCMSP (https://old.tcmsp-e.com/), STITCH (https://stitch.embl.de/) and DrugBank (https://go.drugbank.com/) at their respective official websites.

## Supplementary Materials

The following supporting information can be downloaded at: https://www.mdpi.com/article/10.3390/ph19101595/s1, Table S1: Details of antibodies used in Western blot experiments; Table S2: Primer sequences used for qRT-PCR analysis in lung tissue; Table S3: Primer sequences used for qRT-PCR analysis in HPASMCs; Table S4: Top eight protein-ligand hydrogen bonds ranked by occupancy; Table S5: DCCM clustering results; Table S6: Contact and hydrogen-bonding characteristics of NF-κB DNA recognition interface residues during MD simulation; Table S7: Comprehensive Molecular Docking Parameters, Binding Energies, and Detailed Residue Interactions of SCU with Core Targets; Figure S1: Predicted binding interactions between scutellarin and core inflammatory related target proteins; Figure S2: Time evolution of the radius of gyration (Rg) during the molecular dynamics simulation; Figure S3: Time evolution of protein solvent-accessible surface area (SASA); Figure S4: Time evolution of the minimum ligand-pocket distance (y-axis) and pocket volume (x-axis); Figure S5: Time evolution of ligand rotatable-bond dihedral angles (lower panel) and χ1 flip counts (upper panel); Figure S6: Dynamic cross-correlation matrix (DCCM) of protein Cα atoms. Colors indicate correlation patterns: blue represents positive correlation, and red represents anti-correlation; Figure S7: Phase contrast imaging for phenotypic alteration in human pulmonary artery smooth muscle cells (HPASMCs); Figure S8: SCU suppresses SuHx induced elevation of α SMA in mouse lung tissue; Figure S9: Figure X. SCU reduces F4/80 positive macrophage infiltration in the lungs of SuHx exposed mice; Figure S10: Correlation analysis between inflammatory signaling activation and pathological characteristics in SuHx-induced PAH rats.

## Abbreviations

The following abbreviations are used in this manuscript:

BP	Biological process
CC	Cellular component
DMSO	Dimethyl sulfoxide
DCCM	Dynamic cross-correlation matrix
FEL	Free-energy landscape
GO	Gene ontology
GSDMD	Gasdermin D
GSDMD-N	N-terminal fragment of GSDMD
HE	Hematoxylin-Eosin
HPASMCs	Human pulmonary artery smooth muscle cells
IκBα	Nuclear factor of kappa light polypeptide gene enhancer in B-cells inhibitor, alpha
KEGG	Kyoto Encyclopedia of Genes and Genomes
MF	Molecular function
MIC	minimum-image convention
MMP-9	Matrix metallopeptidase-9
NF-κB	Nuclear factor kappa-B
NIH	National Institutes of Health
NLRP3	NLR family pyrin domain containing 3
PAH	Pulmonary arterial hypertension
PAT	Pulmonary artery acceleration time
PBC	Periodic boundary condition
PCA	Principal component analysis
PEG300	Polyethylene glycol 300
PET	Pulmonary artery ejection time
PPI	Protein-protein interaction
Rg	Radius of gyration
RMSD	Root-mean-square deviation
RMSF	Root-mean-square fluctuation
RVAW	Right ventricular anterior wall thickness
SASA	Solvent-accessible surface area
SCU	Scutellarin
SMILES	Simplified Molecular-Input Line-Entry System
SuHx	Su5416-hypoxia
TAPSE	Tricuspid annular plane systolic excursion
TF	Transcription factor
TNF	Tumor necrosis factor
WT%	Medial wall thickness percentage
WA%	Medial wall area percentage
