# Supplementary material for: Scutellarin Alleviates Pulmonary Hypertension by Inhibiting the NF-κB/NLRP3 Signaling Pathway to Suppress Inflammation and Pyroptosis
Source: Pharmaceuticals (Basel). 2026 Oct 8;19(10):1595. doi: 10.3390/ph19101595 (PMC13708133; doi:10.3390/ph19101595)
Supplement: Supplementary file 1 [file pharmaceuticals-19-01595-s001.zip › Supplementary Figures.pdf]

Supplementary information

Molecular docking of scutellarin with inflammatory-related target proteins

Molecular docking was performed to characterize the binding capability of scutellarin to four key inflammatory-associated targets, IL-18, JUN, TNF, and RELA. As shown in Figure S1, scutellarin could stably occupy the active pocket of each target protein and form hydrogen bonds with critical amino acid residues. For IL-18, scutellarin formed hydrogen bonds with ARG-27; for TNF, hydrogen-bond interaction was observed with GLN-88. Scutellarin established hydrogen-bond contacts with DG-308 and DC-213 on JUN-DNA complex, and interacted with SER-483 of RELA. These findings suggested that scutellarin may exert anti-inflammatory effects by directly binding to these target molecules.

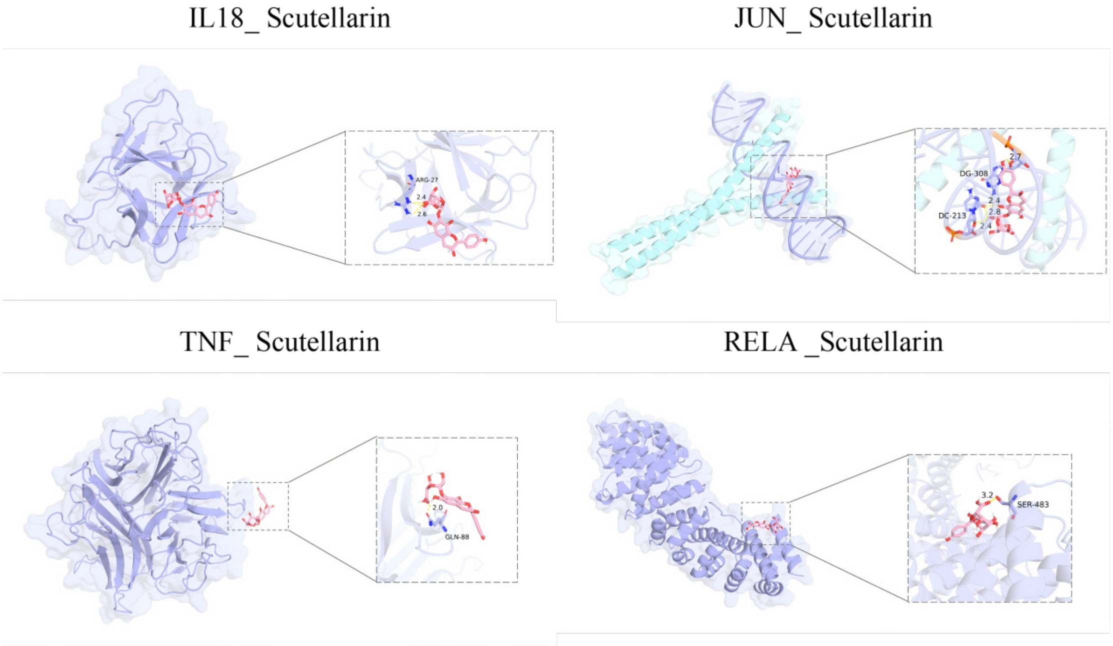

Figure S1. Predicted binding interactions between scutellarin and core inflammatory-related target proteins. Three-dimensional docking diagrams illustrate the spatial binding of scutellarin to IL-18, JUN, TNF and RELA. Enlarged panels highlight critical amino acid residues participating in hydrogen-bond interactions, with numerical values representing hydrogen-bond lengths (Å).

Simulation System and Methodology

The initial structure was derived from the optimal p50 (rep_nfkb.pdb, residues 43–353)-scutellarin complex conformation obtained from previous AutoDock Vina docking. After parameterization using the AMBER14SB protein force field and GAFF2/AM1-BCC

ligand parameters, the system was solvated in explicit TIP3P water with a 10 Å buffer layer, and charge neutralization was achieved with 0.15 M NaCl. The system subsequently underwent sequential energy minimization, NVT equilibration (300 K, 100 ps), and NPT production simulation.

The production phase was performed from 28.1 to 99.9 ns, generating a single 100 ns trajectory containing 719 frames with a 100 ps interval between frames. All geometric analyses were performed according to the minimum image convention (MIC). The ligand was reconstructed under MIC conditions through periodic boundary condition (PBC) unwrapping and nearest-image translation. Protein Cα atoms were aligned to the initial frame at 28.1 ns to eliminate overall translational and rotational motions. RMSD, radius of gyration (Rg), solvent-accessible surface area (SASA), RMSF, distance measurements, and pocket volume analyses were calculated using MDTraj.

Hydrogen bonds were identified using the criteria of a donor-acceptor distance < 3.5 Å and a donor-hydrogen-acceptor angle > 150°. Protein-ligand interaction energies were directly calculated using the actual AMBER14SB/GAFF2-AM1-BCC charges and Lennard-Jones parameters from work/system.xml with a cutoff distance of 1.2 nm. These calculations did not include solvation effects or entropy contributions and were therefore used only for comparative trend analysis.

Dynamic cross-correlation matrix (DCCM) analysis and principal component analysis (PCA) were performed based on protein Cα coordinates. The free energy landscape (FEL) was estimated by Boltzmann inversion of a 14 × 14 histogram projected onto the PC1-PC2 conformational space, with energy values expressed in kJ/mol.

Note: The built-in calculate_binding_energy_mm() function in MD_quick_plot was not used because it produced nonphysical results by assigning a hard-coded charge value of q = +0.5 e to all atoms, resulting in excessive same-charge repulsion among approximately 250,000 atom pairs and an unrealistic interaction energy of $+3.5 \times 10^6$ kJ/mol. Therefore, these values were discarded, and the related files were removed from mdqp/INVALID/.

Additional Molecular Dynamics Results

Backbone RMSD and Radius of Gyration

The mean Cα RMSD of the protein throughout the simulation was 3.02 ± 1.26 Å (range: 0.00–6.19 Å), while the average radius of gyration (Rg) was 25.70 ± 0.29 Å (range: 24.91–26.57 Å) (Figure S1). The RMSD profile exhibited a transient increase to 5–6 Å between 76 and 89 ns, followed by a return to approximately 2 Å. In contrast, Rg remained highly stable throughout the simulation, with no apparent expansion or collapse of the protein structure. Together, these results indicate that the protein maintained its overall folded state and that the simulation system remained structurally stable. The extremely low fluctuation of Rg (coefficient of variation, CV ≈ 1.1%) further excludes the possibility of protein unfolding or large-scale conformational collapse.

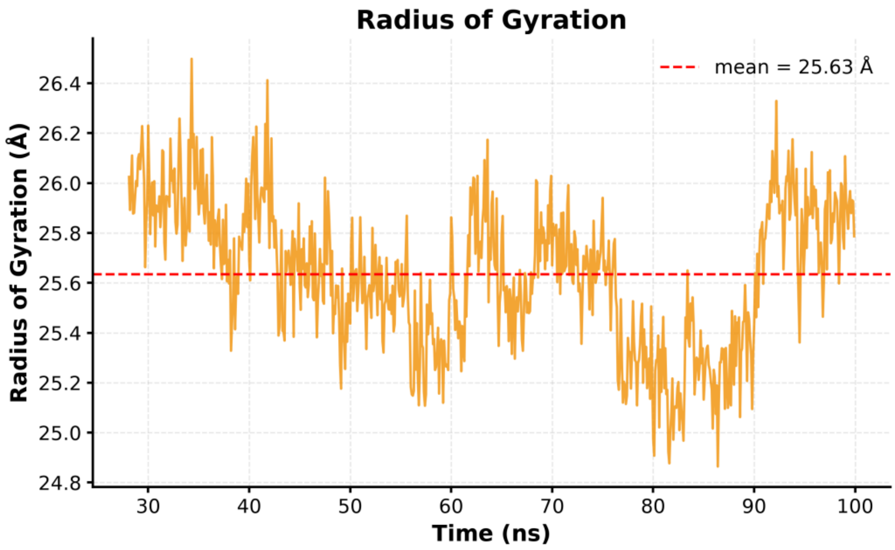

Figure S2. Time evolution of the radius of gyration (Rg) during the molecular dynamics simulation.

Solvent-Accessible Surface Area and Secondary Structure

The solvent-accessible surface area (SASA) of the protein was monitored throughout the entire trajectory, and the results are shown in Figure S2. The average SASA value was 17,090 ± 212 $Å^2$, with a stable profile throughout the simulation. No sustained exposure of previously buried surfaces or formation of newly solvent-accessible regions was observed, suggesting that the complex did not undergo significant conformational dissociation or aggregation.

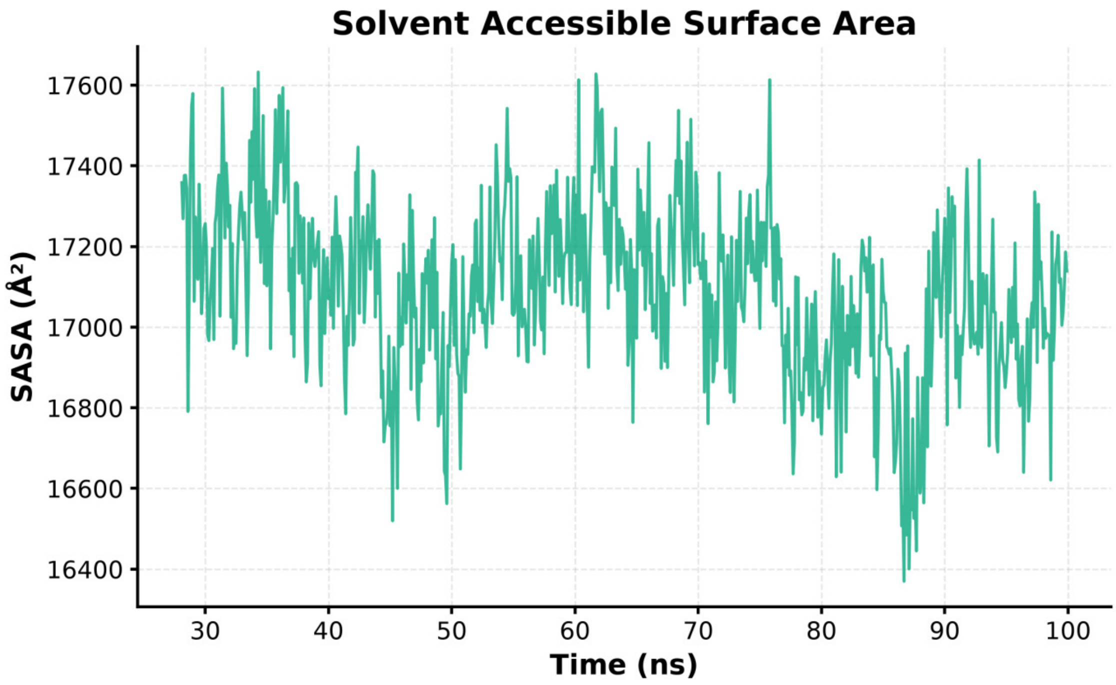

Figure S3. Time evolution of protein solvent-accessible surface area (SASA).

Ligand-Pocket Distance and Pocket Volume

The temporal changes in the minimum ligand-pocket distance and pocket volume were further monitored, and the results are presented in Figure S4. The minimum distance between the ligand and the binding pocket remained at 3.80 ± 0.47 Å throughout the simulation (range: 2.60–5.43 Å). Among all trajectory frames, 69.3% exhibited distances below 4 Å, and 99.2% showed distances below 5 Å, indicating that the ligand maintained close contact with the binding pocket during nearly the entire simulation period.

The pocket volume (approximated as the accessible volume within a 5 Å sphere centered on the ligand) remained stable at 1,586 ± 93 $Å^3$ throughout the trajectory (range: 1,294–1,851 $Å^3$), with only ~6% fluctuation. These results suggest that the binding pocket retained a stable conformation during ligand accommodation, without significant expansion or collapse of the binding interface.

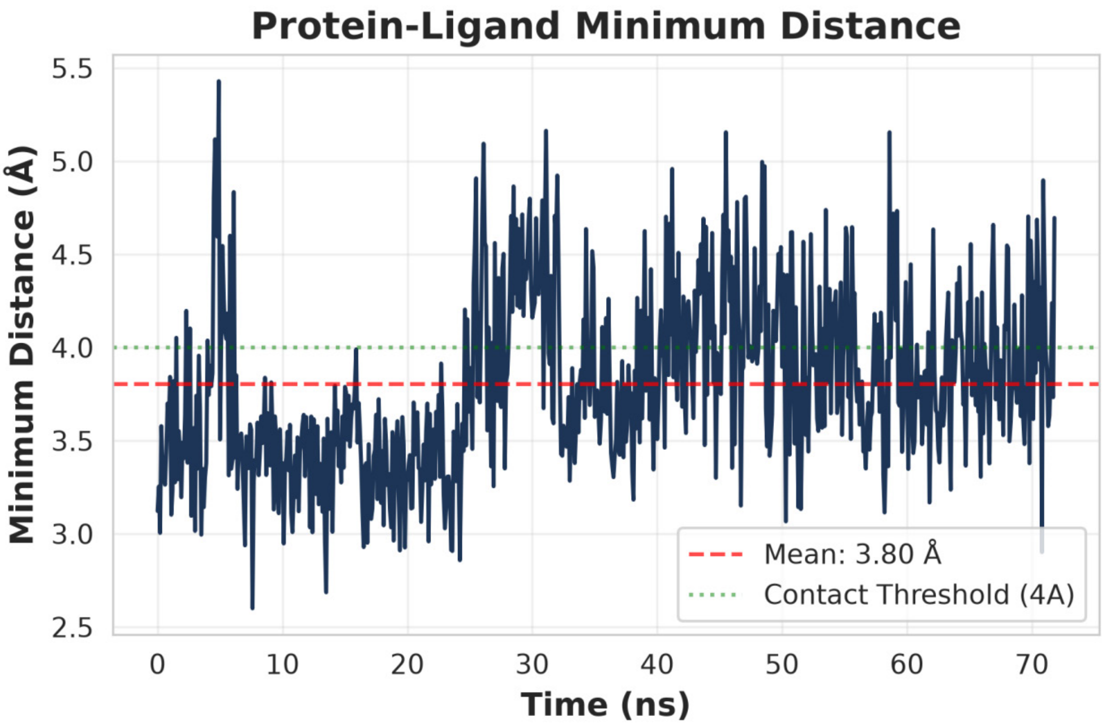

Figure S4. Time evolution of the minimum ligand-pocket distance (y-axis) and pocket volume (x-axis).

Protein-Ligand Interactions

Direct Interaction Energy and Ligand χ1 Dihedral Angle

The direct protein-ligand interaction energy was calculated using the actual AMBER14SB/GAFF2-AM1-BCC force field parameters from *work/system.xml* with a cutoff distance of 1.2 nm, and the results are presented in Figure S6. The electrostatic contribution was −100.22 ± 297.44 kJ/mol (range: −1,080.5 to +797.4 kJ/mol), while the van der Waals contribution was −86.19 ± 12.42 kJ/mol (range: −129.7 to −44.6 kJ/mol). The total direct interaction energy was −186.41 ± 296.98 kJ/mol (range: −1,158.3 to +696.8 kJ/mol).

The van der Waals component exhibited minimal fluctuation (CV ≈ 14%) and represented a stable, low-noise contributor to ligand binding. In contrast, the electrostatic component showed substantial variation (CV ≈ 297%), which is consistent with the characteristic Coulombic fluctuation of flavonoid compounds containing multiple hydroxyl groups within polar binding interfaces. Importantly, these values represent gas-phase direct interaction energies without solvent effects or entropic contributions

and should not be interpreted as binding free energy (ΔGbind). Instead, they were used only to evaluate relative changes in interaction strength during the simulation.

The χ1 dihedral angle flipping profile of ligand-interacting residues (Figure S5) demonstrated that the key binding-interface residues ARG57, ARG59, PHE56, PHE58, and TYR60 exhibited zero χ1 flips throughout the 100 ns simulation. In contrast, peripheral pocket residues, including LYS80 (50 flips), LEU70 (26 flips), ASN139 (22 flips), and ASN250 (19 flips), displayed greater side-chain mobility. These observations are consistent with the residue contact analysis, indicating that the side-chain orientations of core binding residues remained highly stable, whereas residues located at the pocket periphery retained greater conformational flexibility.

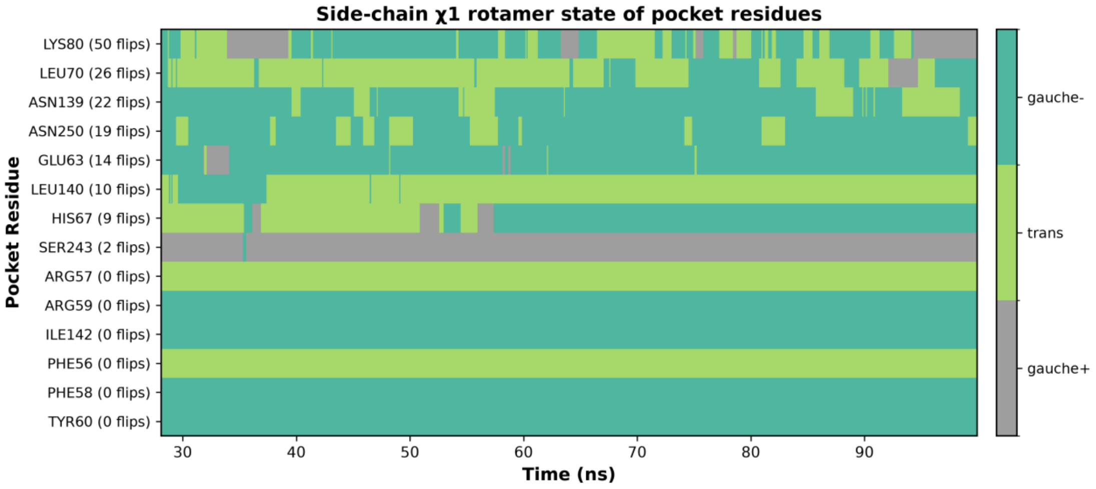

Figure S5. Time evolution of ligand rotatable-bond dihedral angles (lower panel) and χ1 flip counts (upper panel).

Dynamic Cross-Correlation Matrix (DCCM)

The displacement cross-correlations between protein Cα atom pairs were calculated, and hierarchical clustering was performed to identify correlated motion modules. The results are presented in Figure S7 and Supplementary Table 5. A total of six correlated motion clusters were identified. Cluster 1 encompassed loop regions of the DNA-binding domain (residues 49–84, 99–145, and 236–282), representing the major dynamic unit directly interacting with the ligand. Cluster 3 covered the dimerization domain (residues 167–200 and 285–353) and exhibited weak anti-correlated motions with Cluster 1 across

different structural domains. Cluster 6 represented the largest mixed cluster, containing residues 43–48, 125–135, 217–235, 250–272, 315–318, and other regions.

The internal correlation coefficients within Cluster 1 were generally greater than 0.5, indicating that the loop regions of the DNA-binding domain underwent coordinated motions upon ligand binding. These findings provide direct evidence that the DNA recognition surface undergoes collective “breathing” motions during ligand entry into or release from the binding site.

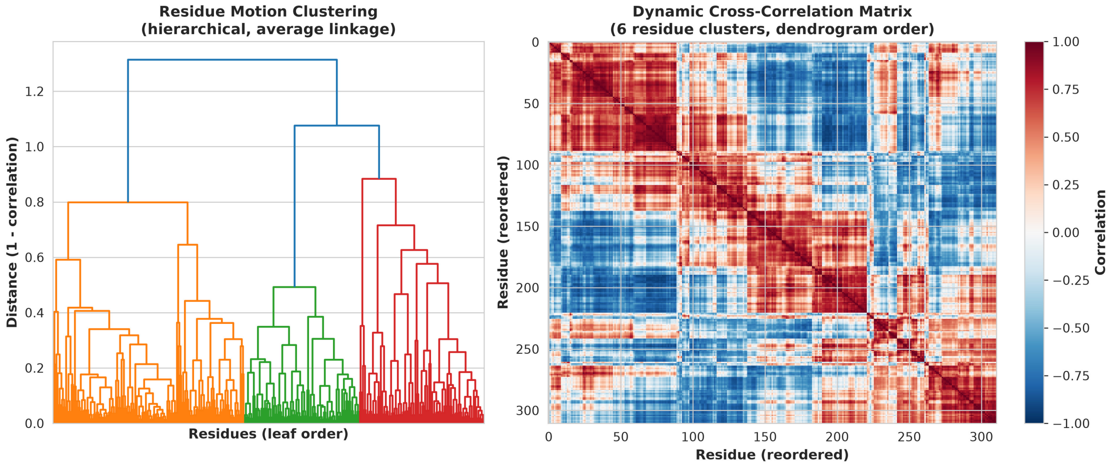

Figure S6. Dynamic cross-correlation matrix (DCCM) of protein Cα atoms. Colors indicate correlation patterns: blue represents positive correlation, and red represents anti-correlation.

Key Findings Associated with NF-κB Inhibition

Key Results

This section reorganizes the aforementioned MD simulation data from the perspective of NF-κB p50 as a potential therapeutic target. The interpretation criteria were defined as follows: (i) **strong-contact residues**: occupancy ≥ 30%; (ii) **persistent hydrogen bonds**: occupancy ≥ 5%; and (iii) **κB DNA recognition interface residues**: p50 residues directly involved in κB DNA binding, including ARG57, ARG59, GLU63, HIS67, ALA62, TYR60, and LYS244. All quantitative values were traced back to the original datasets in *mdqp/TABLES/* and *analysis/*.

Binding Site Localization at the κB DNA Recognition Interface

The experimentally observed binding site during MD simulation (occupancy ≥ 30%) consisted of four core residues: ARG57, ARG59, GLU63, and SER66. Expanding the

criterion to occupancy ≥ 20% identified additional interacting residues, including ARG57, ARG59, GLU63, SER66, HIS67, ALA62, GLY64, and ASN250. Under the broader interaction criterion (occupancy ≥ 30%), a total of 11 interacting residues were identified: ARG57 (100.0%), GLU63 (99.3%), ARG59 (98.1%), HIS67 (52.3%), SER66 (48.5%), ALA62 (42.1%), GLY64 (35.3%), PHE58 (33.0%), GLY55 (32.7%), PHE56 (30.5%), and ASN250 (23.4%). Among these residues, seven (ARG57, GLU63, ARG59, HIS67, ALA62, TYR60, and LYS244) are located within the κB DNA recognition interface, while three residues (ARG57, ARG59, and GLU63) maintained continuous contact throughout 100% of the trajectory frames.

These findings demonstrate that the MD-derived binding site highly overlaps with the DNA recognition interface, and the observed contact frequencies are consistent with residues known to contribute directly to DNA-binding affinity. Among the 11 pocket residues predicted by molecular docking, only two residues (ARG57 and ARG59) retained stable interactions during MD simulation, whereas nine residues (GLY55, PHE56, PHE243, LYS244, ASN249, ASN250, SER274, THR275, and residue 310) were not validated by MD. Conversely, MD analysis identified two additional interacting residues, GLU63 and SER66, that were not predicted by docking.

This comparison indicates that molecular docking primarily represents an initial energetically favorable geometric arrangement, whereas MD simulation reveals the dynamically stabilized binding site. Based on the MD results, the authentic binding interface of scutellarin on p50 is a polar region centered around ARG57/ARG59/GLU63/SER66 within the κB DNA-binding surface, rather than the deeper and relatively hydrophobic pocket predicted by docking.

Arg57 as the Core Residue of the Hydrogen-Bond Network and O8x as a Key Pharmacophore

Among the 31 identified hydrogen bonds, the ligand oxygen atom O8x participated as an acceptor in three major hydrogen-bond interactions, with a combined occupancy of 54.5%: ARG57/NH1···Lig/O8x (31.3%), ARG57/N···Lig/O8x (17.2%), and ARG59/NH1···Lig/O8x (6.0%). ARG57 alone contributed 48.5% of the total

hydrogen-bond occupancy, serving as the central anchor residue of the binding network. Meanwhile, the single ligand atom O8x accounted for more than half of the hydrogen-bond interactions, highlighting the ARG57–ARG59–O8x interaction pair as the core “pharmacophore-target” recognition module between scutellarin and p50.

Consistent with these findings, the ligand-protein $\chi 1$ dihedral angle flipping analysis showed that ARG57, ARG59, PHE56, PHE58, and TYR60 exhibited zero $\chi 1$ flips throughout the 100 ns simulation. The side-chain orientations of these hydrogen-bonding/contact residues remained highly stable during ligand binding, further indicating that the ARG57–ARG59–O8x hydrogen-bond network represents a thermodynamically stable interaction module rather than a transient association dependent on instantaneous conformational fluctuations. This interaction network corresponds to the 4′-OH/5-OH moiety of scutellarin, suggesting that this region represents a key pharmacophore responsible for its NF-κB inhibitory activity.

Electrostatic-van der Waals Coupling and Binding Strength Scale

Decomposition of the direct protein-ligand interaction energy revealed that the van der Waals contribution was −86.19 ± 12.42 kJ/mol (CV ≈ 14%), whereas the electrostatic contribution was −100.22 ± 297.44 kJ/mol (CV ≈ 297%), resulting in a total interaction energy of −186.41 ± 296.98 kJ/mol. Since these values do not include solvation effects or entropic contributions, they should not be interpreted as binding free energy (ΔGbind). However, the calculated interaction energy magnitude is consistent with previously reported direct interaction energies for small molecule-protein complexes (approximately −100 to −300 kJ/mol), suggesting a moderate binding strength.

The relatively stable van der Waals component indicates that the “hard” contact contribution of the complex remains relatively constant after excluding desolvation effects, whereas most fluctuations originate from the electrostatic component. The high coefficient of variation of electrostatic interactions reflects the reversible changes in protonation environment and molecular orientation associated with the five hydroxyl groups and one carbonyl group of scutellarin within the polar binding interface, including indirect interactions mediated by water bridges. This feature is consistent with

the highly charged environment formed by ARG57/GLU63/ARG59, suggesting that binding strength may be sensitive to local microenvironmental factors, such as pH and ionic strength, which should be considered when designing and interpreting in vitro activity assays.

Conformational Sampling: Stable State B1, Transient State B2, and Interface Breathing

The free energy landscape (FEL) analysis identified two major energy basins: B1 (PC1 = −2.27, PC2 = −0.24; 229 frames, 32%) and B2 (PC1 = 5.80, PC2 = 0.42; 121 frames, 17%). B1 persisted throughout the majority of the trajectory (28.1–99.0 ns) and represented the dominant stable conformational state. In contrast, B2 appeared only during 75.9–89.0 ns, corresponding to the RMSD elevation observed between 76 and 89 ns in Figure 3A and the second binding mode identified in Figure 3C (12.9% occupancy, representative frame at 88.7 ns). The free energy difference between B1 and B2 was 0.11 kJ/mol, which is far below RT (2.49 kJ/mol at 300 K), indicating that the difference lacks statistical significance. Nevertheless, the presence of B2 itself is supported by clear trajectory evidence.

DCCM analysis revealed that the loop regions of the DNA-binding domain (Cluster 1; residues 49–84, 99–145, and 236–282) exhibited coordinated motions with internal positive correlations greater than 0.5, which were synchronized with ligand entry and release-associated breathing motions. These results indicate that the κB DNA recognition interface of p50 possesses intrinsic conformational flexibility during scutellarin binding, allowing both prolonged ligand stabilization (long-term residence in B1) and localized conformational transitions (transient sampling of B2). Such interface breathing represents an inherent dynamic property of the NF-κB transcription factor and may directly influence ligand association and dissociation kinetics.

It should be noted that a single 100 ns trajectory represents only one statistically independent sampling event. The B1/B2 free energy difference, which is within the range of 1–2 kJ/mol, is likely influenced predominantly by histogram sampling fluctuations. Specifically, the 719 trajectory frames were projected onto a 14 × 14 grid, resulting in a median of only five frames per bin and a maximum of 24 frames per bin; therefore, the

jagged appearance of the free energy surface should be interpreted as Poisson sampling noise rather than definitive conformational features. More extensive simulations, including free energy perturbation (FEP), thermodynamic integration (TI), enhanced sampling approaches, or multiple independent replicas, would be required to obtain a more accurate free energy landscape and quantitative ligand-binding affinity estimation.

Morphological observation of HPASMCs under different treatment conditions

Phase-contrast microscopy was applied to observe the morphological features of HPASMCs under different experimental conditions. In the HYP group, HPASMCs exhibited typical synthetic-type phenotype with polygonal shape. After scutellarin treatment (HYP+SCU group), cells displayed elongated spindle-shaped morphology, consistent with contractile phenotype. Upon additional TNF-α stimulation (HYP+SCU+TNF-α), HPASMCs reverted to polygonal synthetic-like morphology. These morphological observations indicated that scutellarin could maintain contractile phenotype of hypoxic HPASMCs, while TNF-α partially reversed this protective effect.

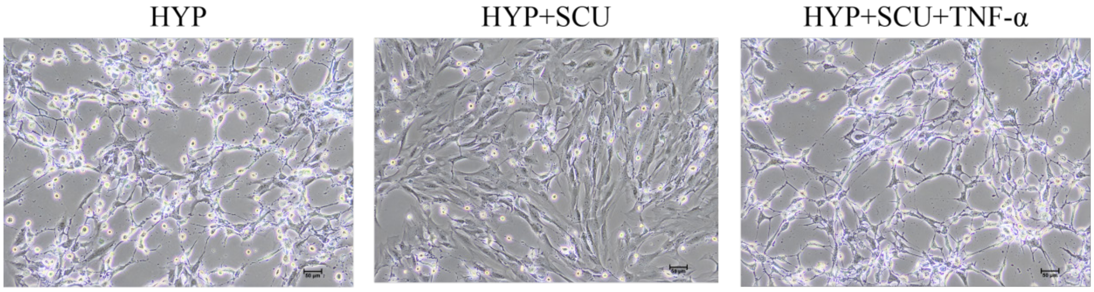

Figure S7. Phase-contrast imaging for phenotypic alteration in human pulmonary artery smooth muscle cells (HPASMCs). Representative phase-contrast micrographs illustrating morphological phenotypes of HPASMCs. HYP group: hypoxia-treated HPASMCs; HYP+SCU group: hypoxia-exposed HPASMCs treated with scutellarin; HYP+SCU+TNF-α group: scutellarin-pretreated hypoxic HPASMCs challenged with TNF-α. Scale bar: 50 μm.

SCU attenuates SuHx-induced upregulation of α-SMA in pulmonary tissues (Figure S8)

Immunofluorescence staining was performed to detect the expression level of α-SMA (red) in lung tissues, and nuclei were labeled with DAPI (blue). Compared with the Ctrl group, the α-SMA-positive staining area in the lung tissues of the SuHx model group was significantly increased ($P < 0.0001$), indicating that SuHx challenge induced pronounced pulmonary vascular muscularization and vascular remodeling. The

α-SMA-positive area in the Vehicle group was comparable to that in the SuHx group. After SCU intervention, the α-SMA-positive staining area was markedly reduced compared with the SuHx/Vehicle group (P < 0.0001). These findings demonstrate that SCU can significantly suppress SuHx-mediated upregulation of α-SMA and alleviate pulmonary vascular remodeling.

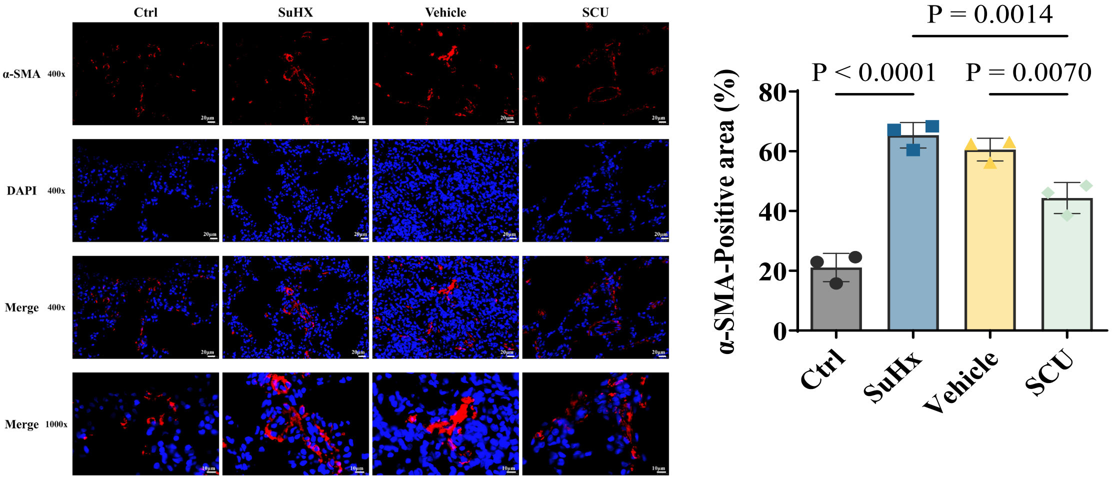

Figure S8. SCU suppresses SuHx-induced elevation of α-SMA in mouse lung tissue. (A) Representative immunofluorescence images of α-SMA (red) and DAPI (blue) in lung sections from Ctrl, SuHx, Vehicle and SCU-treated groups. Scale bar: 20 μm or 50 μm.

SCU inhibits SuHx-mediated pulmonary macrophage infiltration (Figure S9)

F4/80 immunofluorescence staining was performed to label pulmonary macrophages (red), and nuclei were counter-stained with DAPI (blue). The F4/80-positive signals were sparse in the Ctrl group. In the SuHx model group, the number of F4/80-positive macrophages was markedly increased in the pulmonary interstitium and perivascular regions, indicating that SuHx challenge triggered massive macrophage infiltration and pulmonary inflammation activation. The extent of macrophage infiltration in the Vehicle group was comparable to that in the SuHx model group. After SCU intervention, F4/80-positive fluorescent signals were obviously attenuated, accompanied by a prominent reduction in macrophage infiltration. These findings suggest that SCU can effectively suppress SuHx-induced macrophage recruitment and ameliorate pulmonary inflammation in lung tissues.

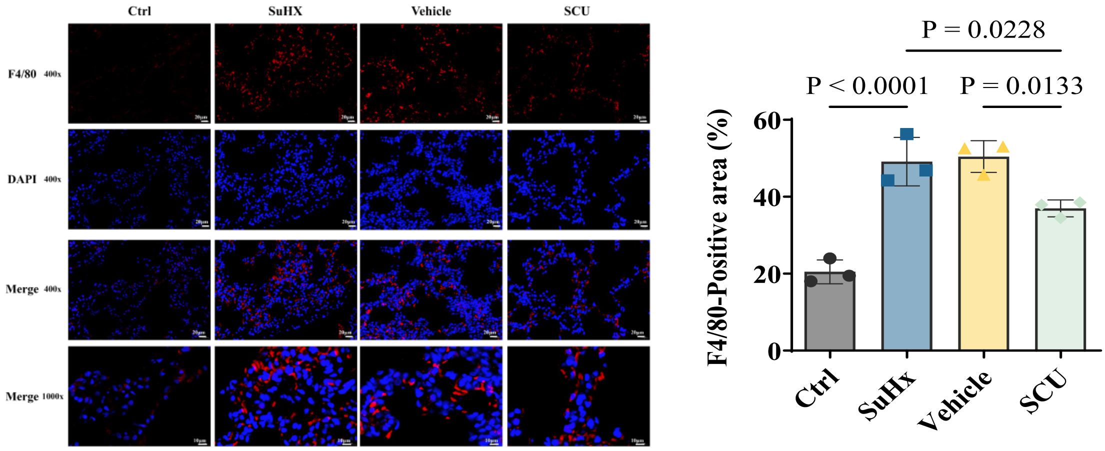

Figure S9. SCU reduces F4/80-positive macrophage infiltration in the lungs of SuHx-exposed mice. Representative immunofluorescence images for F4/80 (red, macrophage marker) and DAPI (blue, nuclear staining) in lung sections from Ctrl, SuHx, Vehicle and SCU groups. Scale bar: 20 μm or 10 μm

Correlation analysis between hemodynamic/pathological parameters and inflammatory signaling markers in SuHx-induced PAH rats

To further investigate the association between pulmonary vascular remodeling, cardiac hemodynamic alterations, and inflammatory signaling activation, correlation analyses were performed between key pathological parameters and molecular markers involved in NF-κB/NLRP3-mediated inflammatory responses. As shown in Figure X, several significant positive correlations were observed between disease severity indicators and inflammatory pathway activation markers. In particular, elevated expression levels of NF-κB-related molecules and NLRP3 inflammasome components were positively associated with increased right ventricular systolic pressure (RVSP), right ventricular hypertrophy index (Fulton index), and vascular remodeling-related parameters. These findings suggest that enhanced inflammatory signaling activation is closely linked to the progression of pulmonary vascular dysfunction and structural remodeling in SuHx-induced PAH. SCU treatment weakened these correlations, indicating that suppression of inflammatory signaling may contribute to its protective effects against PAH progression.

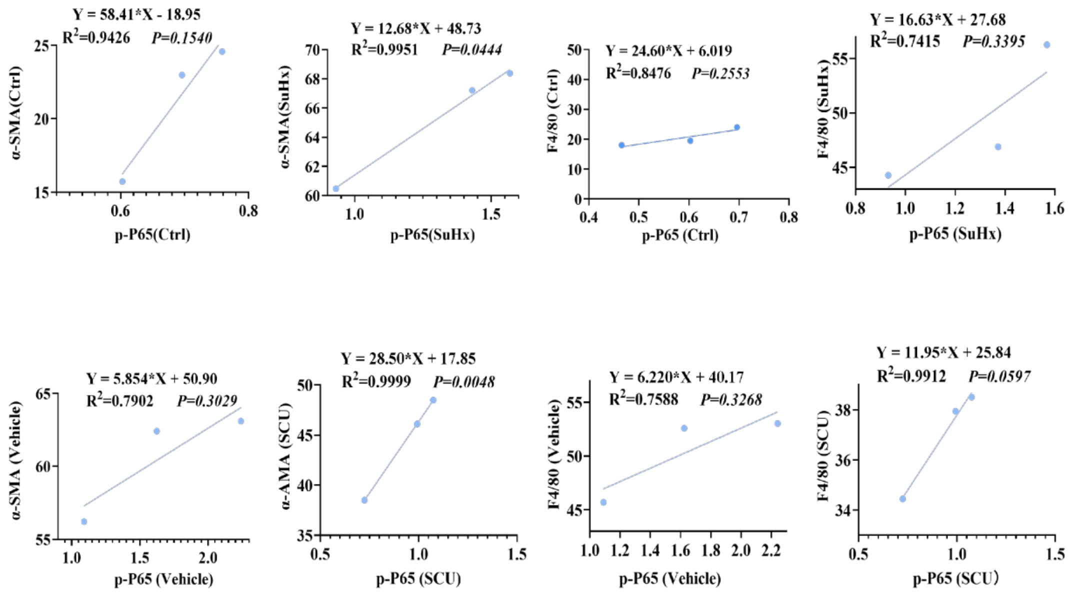

Figure S10. Correlation analysis between inflammatory signaling activation and pathological characteristics in SuHx-induced PAH rats. Pearson correlation analysis was performed to evaluate the relationships between pulmonary hemodynamic parameters, cardiac remodeling indices, histopathological alterations, and inflammatory signaling molecules. Each dot represents an individual animal, and the solid line indicates the linear regression fit. Correlation coefficients (r) and P values were calculated to determine the strength and significance of associations. Data were obtained from individual rats in each experimental group.
