# Supplementary material for: Scutellarin Alleviates Pulmonary Hypertension by Inhibiting the NF-κB/NLRP3 Signaling Pathway to Suppress Inflammation and Pyroptosis
Source: Pharmaceuticals (Basel). 2026 Oct 8;19(10):1595. doi: 10.3390/ph19101595 (PMC13708133; doi:10.3390/ph19101595)
Supplement: Supplementary file 1 [file pharmaceuticals-19-01595-s001.zip › Supplementary Tables.pdf]

Table S1. Details of antibodies used in Western blot experiments

Name	Manufacturer	Catalog No.
IκBa	Proteinetech	10268-1-AP
collagen I	Proteinetech	14695-1-AP
fibronectin	Proteinetech	15613-1-AP
IL-1β	HUABIO	HA601002
IL-18	Affinity	DF6252
NLRP3	Proteintech	30109-1-AP
caspase-1	abmart	M025280
GSDMD	abmart	P30823
GSDMD-N	abmart	P30823s
P65	Affinity	BF8005
p-P65	abmart	TP56372
Vimentin	HUABIO	ET1610-39
MMP9	BIOSS	AF5228
α-SMA	HUABIO	ET1607-53
β-actin	Proteintech	66009-1-Ig
HRP conjugated Goat Anti-Rabbit IgG (H+L)	Servicebio	GB23303
HRP conjugated Goat Anti-Mouse IgG (H+L)	Servicebio	GB23301

Table S2. Primer sequences used for qRT-PCR analysis in lung tissue

Primer		Sequence
IL-1β	F	GACTTCACCATGGAACCCGT
IL-1β	R	GGAGACTGCCCATTCTCGAC
IL-18	F	TCCCCACAACGATGAGTACA
IL-18	R	TTCCTGATCCTGGAGGTTGC
NLRP3	F	AAAGTGCTAAGGACCAGCCC
NLRP3	R	AATGGCTGAAGCTCCCTCAC
caspase-1	F	CACGAGACCTGTGCGATCAT
caspase-1	R	CTTGAGGGAACCACTCGGTC
GSDMD	F	CTATTCAGCCCTCCCGGAAC
GSDMD	R	GCTGTCTGGTATAGTGGGGC
p65	F	GAGAAACAGTGAGGGTTCGGA
p65	R	ACGGGTTAGTCGGTTCAAGC
Vimentin	F	TTTCCAAGCCTGACCTCACC
Vimentin	R	TTAAGGGCATCCACTTCGCA
MMP9	F	ATGGTTTCTGCCCCAGTGAG
MMP9	R	CACCAGCGATAACCATCCGA
Internal reference R-GAPDH	F	GGCCGGAGACGAATGGAAATTA
Internal reference R-GAPDH	R	CCAAATCCGTTCACACCGAC

Table S3. Primer sequences used for qRT-PCR analysis in HPASMCs

Primer		Sequence
IL-1β	F	CCAAACCTCTTCGAGGCACA
IL-1β	R	AGCCATCATTTCACTGGCGA
IL-6	F	TGTGAAAGCAGCAAAGAGGC
IL-6	R	TGAGGGAATGAGGACACACC
TNF-α	F	ATCCTGGGGGACCCAATGTA
TNF-α	R	AAAAGAAGGCACAGAGGCCA
Internal reference H-GAPDH	F	ATGGGCAGCCGTTAGGAAAG
Internal reference H-GAPDH	R	AGGAAAAGCATCACCCGGAG

Table S4. Top eight protein–ligand hydrogen bonds ranked by occupancy (a total of 31 hydrogen-bonding pairs were identified; the complete list is provided in analysis/hbond_occupancy.txt).

Donor	Acceptor	Direction	Occupancy （%）
ARG57/NH1	Lig/O8x	P→L	31.3
ARG57/N	Lig/O8x	P→L	17.2
Lig/O10x	GLY55/O	L→P	6.5
ARG59/NH1	Lig/O8x	P→L	6.0
GLY64/N	Lig/O3x	P→L	5.3
ARG59/NH1	Lig/O9x	P→L	1.8
SER243/OG	Lig/O5x	P→L	1.0
ARG57/NH1	Lig/O7x	P→L	1.0

Table S5. DCCM clustering results (a total of six clusters were identified; the complete residue lists are provided in mdqp/TABLES/scutellarin_p50_dccm_clusters.csv).

Cluster ID	Major residue regions	Domain localization	Representative dynamic features
1	49–84, 99–100, 112–124, 136–145, 211–216, 236–249, 275–282	DNA-binding domain+insert domain	Coordinated breathing motion of loop regions (synchronized with ligand entry and release)
2	101–111, 146–157, 201–210, 250–251, 283–284, 297–298, 334–343	DNA-binding domain+edge region of the dimerization domain	Local flexible motions
3	158–200、261–263、285–296、319–333、344–353	Dimerization domain	Weak anti-correlated motion with Cluster 1
4	252–254, 273–274	N-terminal region of the dimerization domain	Small-scale local motions
5	299–314	C-terminal region of the dimerization domain	Local flexible motions
6	43–48, 89–98, 125–135, 217–235, 255–272, 315–318	DNA-binding domain+N-terminal region of the dimerization domain	Largest mixed cluster with weak inter-domain correlations

Table S6. Contact and hydrogen-bonding characteristics of NF-κB DNA recognition interface residues during MD simulation (data sources: mdqp/TABLES/scutellarin_p50_residue_contacts.csv and analysis/hbond_occupancy.txt)

NF-κB DNA interface residues	MD occupancy （%）	Role in MD	High-occupancy hydrogen bonds （%）
ARG57	100.0	Anchor residue / hydrogen-bond core	31.3 (NH1···O8x) + 17.2 (N···O8x)
GLU63	99.3	Anchor residue / electrostatic interface	—
ARG59	98.1	Anchor residue / hydrogen-bond donor	6.0 (NH1···O8x)
HIS67	52.3	Moderate contact / π-stacking	0.3 (ND1···O8x)
ALA62	42.1	Moderate contact / hydrophobic interaction	—
TYR60	3.6	Weak contact	—
LYS244	1.5	Very weak contact	—

Table S7. Comprehensive Molecular Docking Parameters, Binding Energies, and Detailed Residue Interactions of SCU with Core Targets.

Target Gene	Protein Complex (PDB ID)	Grid Center Coordinates (x,y,z)	Grid Box Dimensions (x,y,z in Å)	Binding Free Energy ΔG (kcal/mol)	Redocking RMSD (Å)	Hydrogen Bonding Residues / Nucleotides	H-Bond Lengths (Å)	Hydrophobic & Non-covalent Interacting Residues
JUN	1JNM	(-2.288, -0.150, -0.893)	45.97, 75.39, 90.11	-9.4	0.92	DG-308, DC-213	2.7 (DG); 2.4, 2.8, 2.4 (DC)	LYS-268, ARG-270, GLY-271
NFKB1	1SVC	(-2.288, -0.150, -0.893)	45.97, 51.49, 33.10	-7.1	1.08	PRO-29	1.8	LYS-30, ARG-54, PHE-56
IL18	1J0S	(-2.288, -0.150, -0.893)	52.17, 39.99, 50.43	-7.1	1.15	ARG-27	2.4, 2.6	GLU-31, LEU-33, LYS-53, PHE-138
RELA	7LEU	(-2.288, -0.150, -0.893)	45.97, 88.27, 82.75	-6.4	1.35	SER-483	3.2	LEU-481, ALA-484, VAL-487
TNF	1A8M	(-2.288, -0.150, -0.893)	62.30, 71.20, 66.75	-4.2	0.88	GLN-88	2	TYR-87, LEU-90, VAL-91
